# Advanced Therapeutic Approach Based on LDHs–Mimetic Oxidoreductase

**DOI:** 10.1002/advs.202519115

**Published:** 2025-11-21

**Authors:** Kai Song, Yu Wei, Peng Jia, Shan He, Qian Wang, Manman Xu, Hongzhi Liu, Pengtao Bao, Shanyue Guan, Xiaozhong Qu

**Affiliations:** ^1^ Technical Institute of Physics and Chemistry Chinese Academy of Sciences Beijing 100190 P. R. China; ^2^ Key Laboratory of Geriatric Nutrition and Health Ministry of Education Technology and Business University 11 Fucheng Road, Haidian District Beijing 100048 P. R. China; ^3^ Department of Geriatrics Guang’ anmen Hospital China Academy of Chinese Medical Sciences Beijing 100053 P. R. China; ^4^ The Eighth Medical Center Chinese PLA General Hospital Beijing 100089 P. R. China; ^5^ College of Materials Science and Opto−Electronic Technology University of the Chinese Academy of Sciences Beijing 101408 P. R. China

**Keywords:** LDHs−based nanozyme, pathological microenvironment, redox process

## Abstract

Redox dysregulation is recognized as a key driver in the pathophysiology of numerous refractory diseases, contributing significantly to the progression and poor prognosis. The precise targeting and sophisticated modulation of redox processes within pathological microenvironments thus offer a promising avenue for innovative therapeutic strategies. Layered double hydroxides (LDHs)−based nanozyme lies in the programmable multi−active site architecture, which enables unprecedented functional integration for precise pathological microenvironment remodeling. In this review, the redox modulating mechanisms of LDHs−based nanozyme in these critical disease contexts are systematically explored, with special emphasis on intrinsic enzyme−like activities and structure−activity relationships. At the same time, it highlights how designing LDHs−based nanozyme can manipulate redox homeostasis to precisely reprogram the pathological microenvironment for stimulating effective, context−dependent pro− and anti−inflammatory therapeutic outcomes, which is a crucial requirement in conditions such as tumors and tissue injury. Finally, building upon recent advances, a forward−looking perspective is provided on the current challenges and future research directions in this rapidly progressing field.

## Graphical abstract

Overview of Sructure, Multienzyme‐mimicking Activity and Advanced Therapeutic Approach Based on LDHs–Mimetic Oxidoreductase.

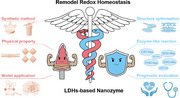

## 1 Introduction

The pathological microenvironment, characterized by distinct biochemical and physiological alterations in local tissues during disease progression, plays a decisive role in inflammatory regulation and therapeutic outcomes. Unlike normal physiological conditions, this dysregulated milieu typically features abnormal redox homeostasis, dysregulated pH, and altered cellular composition, collectively creating a self−sustaining cycle that drives disease advancement. At the molecular level, these microenvironments exhibit excessive accumulation of reactive oxygen species (ROS) and reactive nitrogen species (RNS), coupled with dysfunctional antioxidant defense systems, establishing a persistent state of oxidative stress that critically influences both pro− and anti−inflammatory responses. The complex interplay between various cell types, bioactive molecules, and extracellular matrix components within this environment creates unique spatiotemporal dynamics that conventional therapeutic approaches often fail to address effectively. As representative models of pro− and anti−inflammatory conditions, tumor microenvironments (TME) and tissue injury sites exemplify these challenges, where precise modulation of redox balance emerges as a promising therapeutic strategy. This review will focus on discussing innovative therapeutic approaches that target these pathological microenvironments, with particular emphasis on the emerging role of nanozyme−based for restoring redox homeostasis.

Generally, the pathological microenvironment can be categorized into two types, oxidative− and redutive−type, each with distict inflammatory profiles. The TME represents the oxidative type, characterized by hypoxia, acidic pH, elevated ROS, and a paradoxical mix of pro− and anti−inflammatory signals that promote tumor growth and suppress anti−tumor immunity.^[^ ^1^ ^]^ As an ecosystem closely intricately linked to tumor initiation and progression, TME is constituted with cancer cell, immune cell, stromal cell and other components.^[^ ^2^ ^]^ To survive in the high−stress microenvironment associated with excessively elevated ROS levels, cancer cells significantly upregulate antioxidant capacity.^[^ ^3^ ^]^ This adaptive response maintains ROS at a high yet controllable state, enabling a delicate balance between proliferation and survival. It is this distinctive redox homeostasis that represents a promising therapeutic target in oncology. Conversely, the redutive−type is typified by the tissue injury, especially ischemia−reperfusion injury (IRI) microenvironment, which triggers a cascade of oxidative stress, neuroinflammation, Ca^2+^ over loaded, etc.^[^ ^4^, ^5^, ^6^ ^]^ For the IRI microenvironment, after the restoration of blood supply to brain tissue following an ischemic stroke (IS) or myocardial ischemia–reperfusion (MIR) injury,^[^ ^7^, ^8^, ^9^ ^]^ tissue damage occurs, accompanied with increased neuronal death through protein oxidation,^[^ ^10^ ^]^ DNA damage,^[^ ^11^ ^]^ and lipid peroxidation.^[^ ^12^ ^]^ This represents a highly dynamic pathological process characterized by two distinct phases. The initial ischemic phase creates a severely oxygen deprived environment where metabolic waste and reductive substances accumulate. This is abruptly followed by the reperfusion phase, where sudden oxygen reintroduction triggers rapid and massive ROS production, leading to extreme oxidative stress. This can be attributed to excessive generation of ROS, such as superoxide anion radicals (•O_2_^−^),^[^ ^13^ ^]^ hydroxyl radicals (•OH),^[^ ^12^ ^]^ and hydrogen peroxide (H_2_O_2_).^[^ ^14^ ^]^ If these ROS are not captured after vascular recanalization, the prognosis after IS will not be improved.^[^ ^15^, ^16^ ^]^ Although TME and IRI microenvironments exhibit distinct pathological mechanisms, both share redox homeostasis imbalance, initiating a robust inflammatory reaction.^[^ ^17^, ^18^ ^]^ Understanding these distinct redox landscapes is crucial for developing effective nanozyme based therapies, as it highlights the need for materials that can adapt to different oxidative stress patterns and manage varying redox challenges across different disease contexts. To fulfill these demands, the regulation of redox processes in the pathological environment is the key issue for achieving advanced tumor and cerebrovascular diseases treatment.

As a neoteric type of nano−reagent with enzyme−mimicking characteristics, nanozyme endow an appropriate platform for tailing more innovative approaches in the biological filed. Since Yan's group first reported the peroxidase (POD)−like activity of Fe_3_O_4_ nanoparticles,^[^ ^19^ ^]^ various nanozymes have been discovered with the capability of mimicking the natural enzymes.^[^ ^20^, ^21^, ^22^ ^]^ Compared to natural enzyme, nanozyme is possessed of higher stability, designable, multifunctional, and targeting property, etc.^[^ ^23^ ^]^ Especially, nanozyme that comprises redox−active metals is highly needed due to the capability to in situ interfere catalytic reactions and adjust subtle homeostasis in the pathological microenvironment. Specifically, most nanozymes are reported to mimic the redox properties of natural enzymes,^[^ ^24^, ^25^ ^]^ mainly including oxidase nanozyme−like activity: POD−,^[^ ^26^ ^]^ oxidase (OXD)−^[^ ^27^ ^]^ and superoxide dismutase (SOD)−like^[^ ^28^ ^]^ activities.^[^ ^29^ ^]^ For example, down−regulating the excess ROS in the organism through the SOD−like activity, promoting the relieving of disorders associated with inflammation.^[^ ^30^ ^]^ In addition, the produced oxygen (O_2_) through catalase (CAT) −like activity can relieve the hypoxic microenvironment in TME, enhancing the curative effect of therapy. The OXD− and POD−like activities of nanozyme can upregulate the concentration of ROS in organisms, which is beneficial in the tumor treatment by inducing oxidative stress to trigger apoptosis.^[^ ^31^ ^]^ It is noteworthy that, distinct from natural enzyme, nanozyme frequently exhibits multi−enzyme characteristics. This property enables it not only to facilitate cascade catalytic reactions but also to perform sophisticated catalytic processes within pathological microenvironments. The integration of multiple enzyme−mimetic activities within a single nanozyme platform enables sophisticated catalytic cascades that significantly enhance therapeutic efficacy. A prominent example lies in the combination of SOD− and CAT−like activities, which establishes a complete ROS elimination pathway. This system first catalyzes the dismutation of •O_2_^−^ into H_2_O_2_, then immediately decomposes H_2_O_2_ into harmless H_2_O and O_2_, thereby achieving comprehensive ROS clearance while preventing the accumulation of toxic intermediates. Another strategically designed cascade couples glucose oxidase (GOx)−like activity with POD−like function, creating a self−amplifying therapeutic system. The GOx−like component consumes intratumoral glucose to generate H_2_O_2_, which subsequently serves as the substrate for the POD−like component to produce highly cytotoxic •OH, resulting in synergistic and context−specific therapeutic outcomes. These orchestrated multi−enzyme systems demonstrate superior efficiency compared to single−enzyme mimics or physically mixed enzymes, as they minimize intermediate diffusion losses and enable spatially confined reaction cascades. The programmable nature of LDHs−based nanozyme makes them particularly suitable for implementing such complex catalytic networks, allowing precise tuning of individual activities and their spatial organization to match specific pathological conditions. Consequently, this significantly enhances the efficiency of redox homeostasis regulation. Therefore, the development of nanozyme with multiple enzyme activities is of great significance.

**Figure 1:**
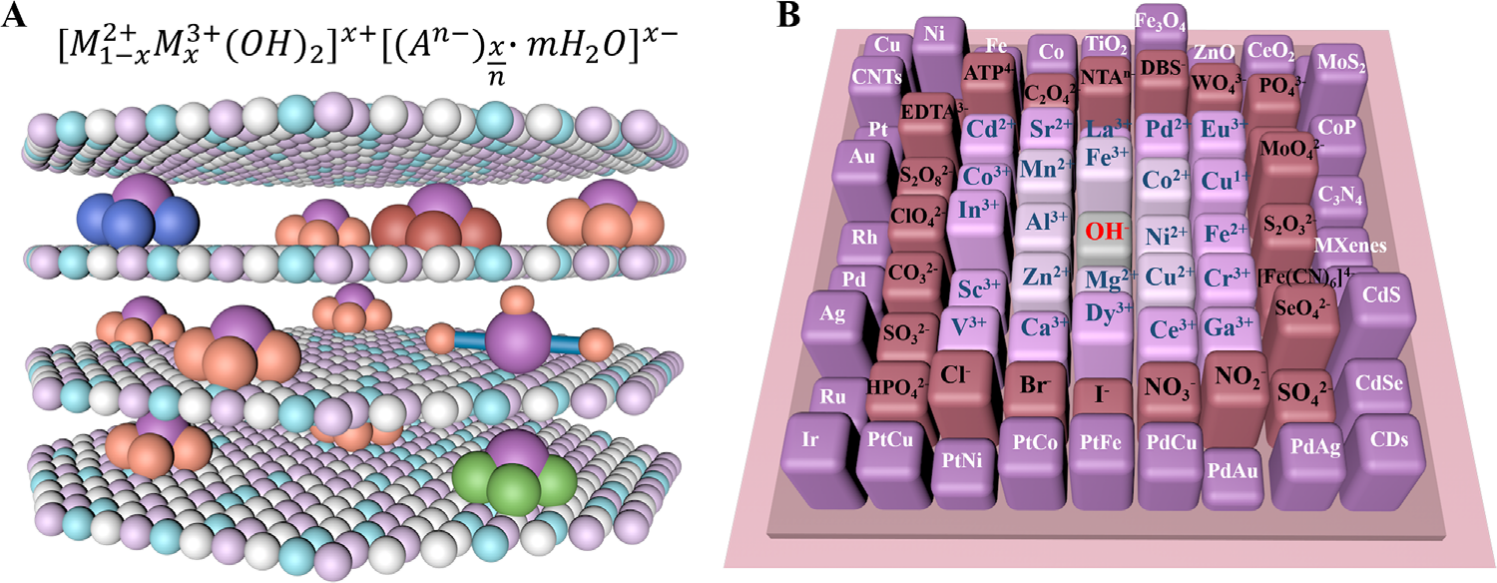
Schematic illustration of the LDHs structure and its “programmable typesetting” design. This figure elucidates the concept of LDHs as a modular nanomaterial platform inspired by typesetting principles. The structure features an open framework that enables precise design and integration of functional “material type blocks” across three core dimensions: the host layer, the interlayer space, and the surface. Similar to assembling movable type, this programmability allows researchers to “print” advanced functional materials with tailored properties.

LDHs known as hydrotalcite or double−layered hydrotalcite, is a type of cationic hydroxide with a layered structure.^[^ ^32^, ^33^, ^34^, ^35^ ^]^ LDHs possess many properties, including biodegradability,^[^ ^36^ ^]^ good biocompatibility,^[^ ^37^ ^]^ high surface−to−volume ratio,^[^ ^38^ ^]^ low cytotoxicity^[^ ^39^ ^]^ and easy delamination or exfoliation.^[^ ^40^ ^]^ As a 2D layered nanozyme, LDHs−based nanozyme is an excellent template for the construction of multifunctional system due to its adjustable geometric and electronic structure^[^ ^41^ ^]^ (**Figure** **1**A). Typically, LDHs−based nanozyme is made up of two metal cations, divalent and trivalent, in the form of double layers or layered structures. Anions and water molecules essentially fill the interlayer gaps between the metal hydroxide layers.^[^ ^42^ ^]^ The general formula for LDHs−based nanozyme is ${\left[M_{1-x}^{2+}M_{x}^{3+}{\left(OH\right)}_{2}\right]}^{x+}{\left[{\left(A^{n-}\right)}_{\frac{x}{n}}\cdot mH_{2}O\right]}^{x-}$, where *M^2+^* and *M^3+^* are the divalent (such as Mg^2+^, Ni^2+^, Zn^2+^, etc.) and trivalent (such as Al^3+^, Fe^3+^, Co^3+^, etc.) metal cations, respectively. A^n−^ is the interlayer anion (such as CO_3_^2−^, NO_3_^−^, SO_4_^2−^, etc.); and *x* is the ratio of M^3+^/(M^2+^ + M^3+^), where 0.17< *x* <0.33.^[^ ^43^, ^44^, ^45^, ^46^, ^47^, ^48^ ^]^

Inspired by movable type printing, which was invented by Bi Sheng during the Northern Song Dynasty using engraved, fired, and assembled ceramic type pieces, this review conceptualizes LDHs−based nanozyme through a “programmable typesetting” design (Figure 1B). Specifically, similar to this historical technique that significantly improved efficiency over woodblock printing, LDHs−based nanozyme comprise host layers that act as a printing plate, with exchangeable ions serving as modular movable type units, allowing nearly unlimited functional combinations. First, the host layer serves as the printing plate. The innermost section consists of covalently bonded metal cations and hydroxyl groups (OH–M^2+^/M^3+^), forming the foundational platform. By selecting different metal ion “type blocks”, such as Mg–Al, Zn–Fe, or Ni–Co, properties including acidity, redox behavior, or magnetism can be precisely tailored. Second, the interlayer space acts as the ink. This intermediate region contains A^n−^ and H_2_O, analogous to ink in printing. Its expansive capacity allows the incorporation of diverse functional “ink units”, ranging from inorganic ions and organic molecules to biomolecules and polyoxometalates, via ion exchange, thereby defining the core functionality of the material. Thirdly, surface modification functions as gilding.^[^ ^49^ ^]^ The outermost layer supports nano−objects, such as noble metal clusters, alloy nanoparticles, or metal oxides, comparable to the decorative gilding applied to printed materials. This step introduces advanced properties, including high electrical conductivity and specific catalytic sites, enabling enhanced functionality through synergistic effects. The programmable nature of LDHs−based nanozyme enables precise control over their electronic structure and catalytic behavior through strategic compositional and structural modifications. By tuning the M^2+^/M^3+^ cation ratio and identity, such as Mg−Al, Co−Fe, or Mn−Fe combinations, the local coordination environment and electron density distribution can be systematically adjusted. Concurrently, structural engineering approaches, including interlayer anion exchange, vacancy creation, and hybridization with functional nanomaterials, further modulate charge transfer properties and active site accessibility. These tailored modifications directly influence critical catalytic parameters, including the adsorption energy of ROS substrates and the energy barriers of key redox reactions, thereby fine tuning enzymatic selectivity and activity. For instance, CoFe−LDHs with optimized cation ratios exhibit enhanced POD‐like activity due to facilitated electron transfer between metal centers, while Mn rich LDHs demonstrate superior SOD‐like capacity owing to their tailored redox cycling capability. These structure activity correlations, validated by spectroscopic studies and theoretical calculations, underscore the role of LDHs as a design platform for customizing nanozyme functions. Regarding the existing published reviews on LDHs−based materials in biology fields, such as bone repair,^[^ ^50^, ^51^ ^]^ there is still a lack of sufficient information and comparison aim at controlling the pathological microenvironment to achieve enhanced redox homeostasis.

**Figure 2:**
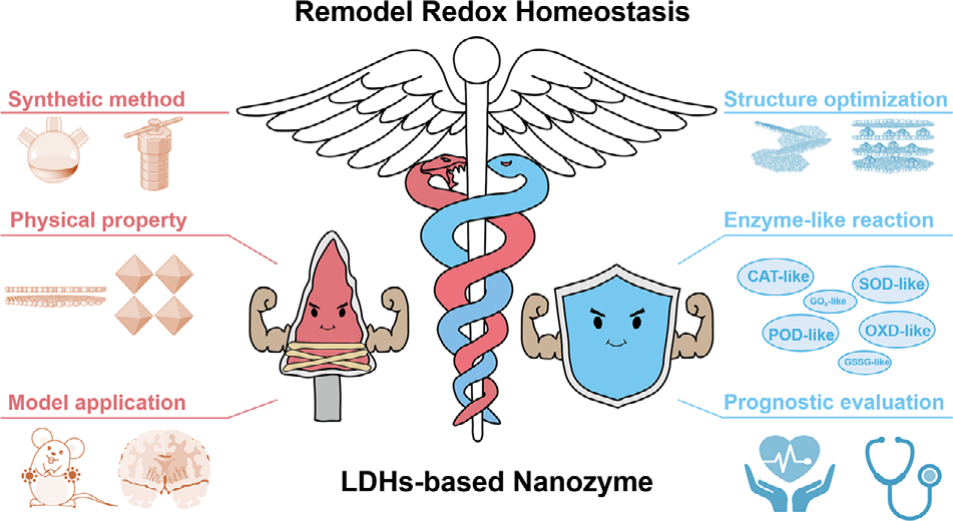
Overview of LDHs−based nanozyme. LDHs−based nanozyme is a class of 2D materials whose chemical composition and surface properties can be precisely tailored by adjusting the ratios of metal ions and interlayer anions, which is widely used for modulating redox pathological microenvironments. Primary synthesis methods include co−precipitation, hydrothermal synthesis, and ion exchange. By mimicking multiple enzyme activities, LDHs−based nanozyme can effectively scavenge or generate excess ROS to alleviate or trigger oxidative stress, and load drugs for synergistic therapy. Both in vitro and in vivo experiments demonstrate the ability to achieve multi−dimensional regulation of the redox microenvironment.

Herein, we envision conducted a systematic review to summarize and prospect LDHs−based nanozyme employed in the reprogramming the pathological microenvironment through remodeling redox homeostasis (**Figure** **2**). This review comprehensively examines recent strategies for modulating and optimizing the LDHs−based nanozyme properties, as well as the unique advantages in remodeling redox homeostasis, particularly in tumor and tissue injury. Initially, we will discuss the conventional hydrothermal synthesis of LDHs,^[^ ^52^ ^]^ ultrathin structural characteristics,^[^ ^53^ ^]^ exfoliation^[^ ^54^ ^]^ and intercalation techniques,^[^ ^55^ ^]^ and utility as precursors for mixed metal oxides (MMOs). Subsequently, we provide a systematic elucidation of the multi−enzyme mimic activities and the application in remodeling redox homeostasis. In conclusion, we present a synthesis and in−depth discussion of contemporary advancements and outcomes in LDHs−based nanozyme research, while addressing the growing interest in the potential for reprogramming the pathological microenvironment.

## 2 Synthesis Strategy of LDHs−Based Nanozyme

There has been various procedures can be applied to synthesize LDHs−based nanozyme, among which co−precipitation is the most widely used and straightforward bottom−up strategy.^[^ ^56^ ^]^ This approach offers strong universality, being applicable to a wide range of divalent and trivalent metal ion combinations, and enables the intercalation of diverse inorganic anions as well as organic macromolecules into the interlayer galleries.^[^ ^57^ ^]^ Typically, aqueous solutions of metal salts in a stoichiometric ratio are introduced into a reactor under vigorous stirring. By maintaining the pH within a basic range of 7–10, co−precipitation of metal ions is induced, leading to the formation of LDHs−based nanozyme. The advantages of this method include high crystallinity of the product, tunable metal composition, and controllable nanosheet dimensions.^[^ ^58^ ^]^ However, inherent limitations such as tendency toward particle agglomeration and relatively low specific surface area may restrict its broader application.^[^ ^59^ ^]^ To overcome these limitations, the hydrothermal method has been widely adopted. In this process, the precursor or reaction mixture is subjected to crystallization under elevated temperature and pressure.^[^ ^60^ ^]^ The hydrothermal system provides a relatively uniform reaction environment, enabling efficient mass and heat transfer, which promotes homogeneous nucleation and crystal growth.^[^ ^61^ ^]^ The high−temperature and −pressure conditions much enhance ion migration and diffusion rates, allowing the precursors to fully dissolve, reorganize, and undergo oriented crystallization. As a result, the hydrothermal method typically yields LDHs−based nanozyme with uniform particle size, improved dispersibility, high crystallinity, and well−defined hexagonal morphology, characteristics that represent the most desirable and ideal form of LDHs−based nanozyme. In addition, other well−established methods such as electrodeposition^[^ ^62^ ^]^ and mechanochemical synthesis^[^ ^63^ ^]^ (e.g., ball milling^[^ ^64^ ^]^) are also commonly employed for the preparation of LDHs−based nanozyme, via a detailed discussion of these techniques is not provided here.

### 2.1 Exfoliation and Intercalation of LDHs−Based Nanozyme

**Figure 3:**
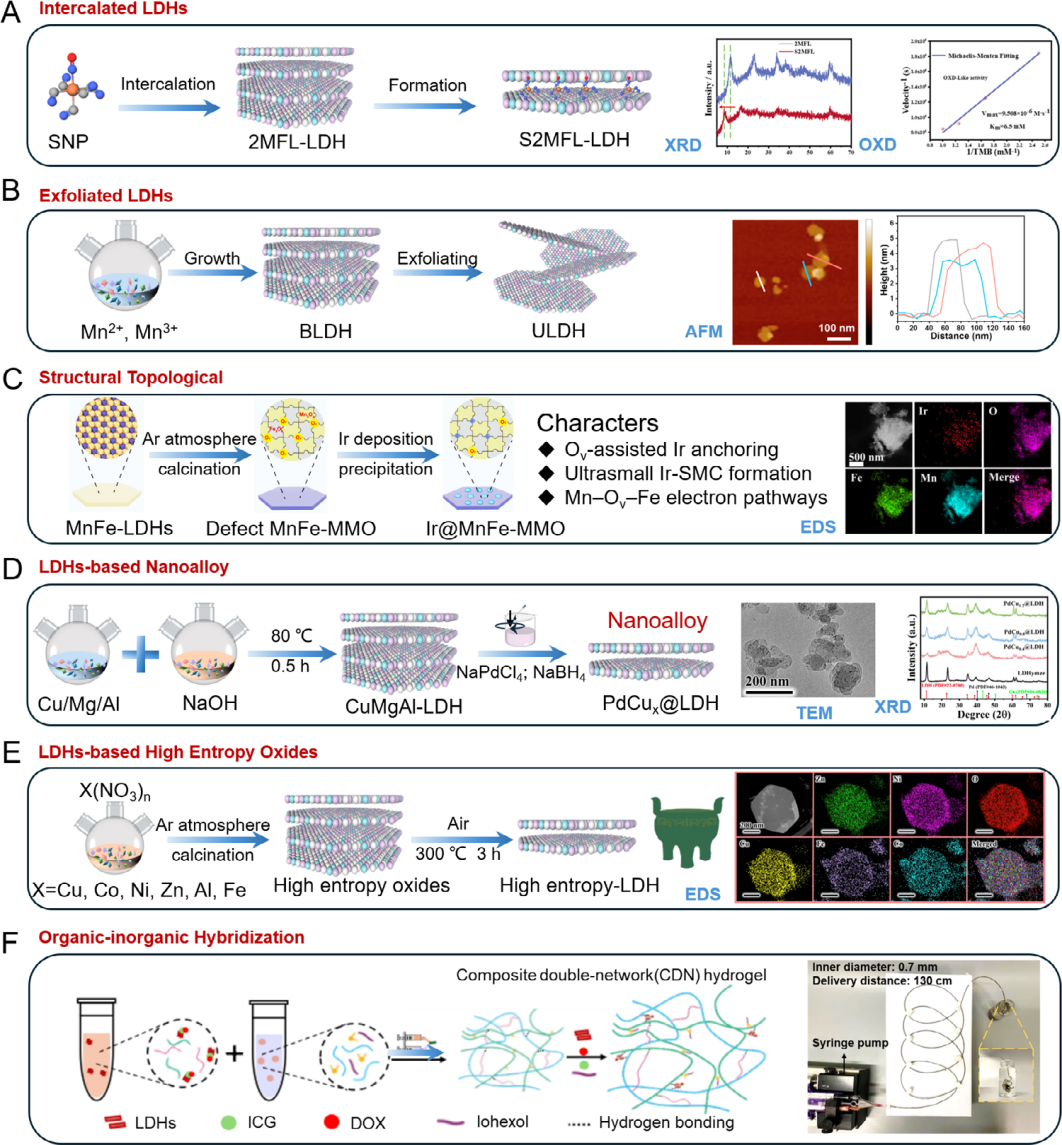
Schematic diagram illustrating synthesis strategies for LDHs−based nanozyme. A) Hydrothermal precipitation for highly crystalline nanoplates. Reproduced (Adapted) with permission. Reproduced (Adapted) with permission.^[^ ^66^ ^]^ Copyright 2022, American Chemical Society. B) Defect engineering to modulate active sites. Reproduced (Adapted) with permission.^[^ ^67^ ^]^ Copyright 2022, Elsevier. C) Intercalation of interlayer species. Reproduced (Adapted) with permission.^[^ ^68^ ^]^ Copyright 2025, Elsevier. D) Calcination−reduction derived oxides. Reproduced (Adapted) with permission.^[^ ^69^ ^]^ Copyright 2024, American Chemical Society. E) LDH−based high−entropy oxides. Reproduced (Adapted) with permission.^[^ ^70^ ^]^ Copyright 2025, American Chemical Society. and F) Organic−inorganic hybridization. Reproduced (Adapted) with permission.^[^ ^71^ ^]^ Copyright 2024, American Chemical Society.

The exfoliation and intercalation have received much attention, owing to the critical role in producing atomically thin sheets and multilayered structures with surface charges of LDHs.^[^ ^65^ ^]^ Intercalation, refering to the process of introducing foreign molecules, ions, or functional groups into the interlayer region of LDHs−based nanozyme through physical or chemical means without disrupting the original layered host structure.^[^ ^55^ ^]^ Guo et al.^[^ ^66^ ^]^ through a supramolecular intercalation strategy (**Figure** **3**A), nitroprusside (SNP) anions were successfully incorporated into MgMnFe−LDH interlayers, forming a stable and functional nanoagent denoted as S2MFL. This host–guest architecture not only enhances the stability and delivery efficiency of SNP but also endows the system with triple enzyme−mimetic activities under TME stimulation. The resulting cascade catalytic reactions enable simultaneous generation of ROS and RNS, significantly amplifying oxidative stress and breaking redox homeostasis in tumor cells. This approach provides a robust and synergistic platform for enhanced chemodynamic therapy and offers a promising strategy for TME responsive theranostics.

Exfoliation, on the other hand, involving the complete separation of the multilayer structure of LDHs into individual or a few layers.^[^ ^54^ ^]^ This process typically begins with intercalation of bulky guest species to expand the interlayer spacing. Exfoliation is then achieved through external forces, such as mechanical agitation or ultrasonication, or through solvent interactions that overcome interlayer electrostatic and van der Waals forces.^[^ ^72^ ^]^ Notably, interlayer hydrogen bonding is the primary factor responsible for the tightly packed LDHs layers.^[^ ^73^ ^]^ The exfoliation of layered materials typically involves a two−step process. Initially, macromolecules are employed to intercalate the layered material, thereby expanding the interlayer spacing, followed by an exfoliation step to obtain isolated nanosheets.^[^ ^74^, ^75^ ^]^ However, the dispersion of nanosheets obtained through exfoliation tends to be unstable, as they can readily self−assemble to restore the layered structure of LDHs. Consequently, various effective methods have been developed to prepare LDHs nanosheets. Wang et al.^[^ ^67^ ^]^ developed a method for preparing ultrathin MgMn−LDH (ULDH) (Figure 3B). The process begins with the synthesis of bulk LDH (BLDH) through oxidation coprecipitation. The as−prepared BLDH was dispersed in N,N−dimethylformamide (DMF) and subjected to alternating probe and bath sonication for 8 h under N_2_ atmosphere. Subsequently, gradient centrifugation was performed: first at 3000 rpm to remove unexfoliated aggregates, followed by 9000 rpm to collect the ultrathin nanosheets. This procedure yielded ULDH nanosheets with a thickness of ≈4 nm, which well preserve their bioactive sites, making them promising for applications in spinal cord injury therapy.

### 2.2 Vacancy Engineering and Structural Topological of LDHs−Based Nanozyme

Recent studies have revealed that in catalytic reactions, the role of active sites extends beyond simple surface locations, often concentrating at interfacial sites. This is primarily because interfacial structures can induce unique geometric and electronic configurations, as well as modulate the functional properties of active components. Defect engineering^[^ ^76^ ^]^ enables precise modulation of the electronic structure and surface properties of LDHs through the intentional introduction of oxygen vacancies (O_v_),^[^ ^77^ ^]^ metal vacancies,^[^ ^78^ ^]^ heteroatom doping,^[^ ^79^ ^]^ etc. These defects can be served as active sites that appreciably enhance the electrical conductivity of LDHs, optimizing the adsorption and activation of reactant molecules, and improving the overall catalytic performance.^[^ ^80^ ^]^ As a catalyst precursor, LDHs allow facile adjustment of both compositional types and ratios according to specific requirements. Moreover, thermal topological transformation stands as one of the most critical characteristics of LDHs.^[^ ^81^ ^]^ Structural topotactic transformation leverages the well−ordered cationic arrangement in the layers of LDHs as a parent template. Through precisely controlled topochemical reactions, such as thermal calcination,^[^ ^82^ ^]^ hydrothermal etching,^[^ ^83^ ^]^ or anion exchange,^[^ ^84^ ^]^ this approach allows fine−tuning of the electronic structure and phase composition while preserving the macroscopic morphology and metal species of the precursor.^[^ ^85^ ^]^ During thermal treatment, reducible components migrate predominantly in a vertical direction, leading to the formation of abundant interfacial sites. These sites play an essential role in enabling efficient catalytic processes by providing a highly reactive environment that facilitates the activation of reactants and intermediates, thereby enhancing both catalytic efficiency and selectivity. However, the current approach still faces challenges in achieving uniform defect distribution and precise control over metal−support interactions, particularly when scaling up the synthesis process for biomedical applications.

The close encapsulation of metal nanoparticles by metal oxides induces strong metal–support interactions, encompassing effects such as electron transfer and spatial confinement.^[^ ^86^ ^]^ By introducing reducible metal cations into the LDHs layers, the migration and aggregation of metal oxides (MO_x_) during interface construction can be effectively controlled via topological regulation. Such uniquely structured interfaces contribute substantially to reactant orbital matching, enhanced adsorption strength/modes, and multifunctional catalytic performance. Consequently, LDHs−based materials regarded as a highly advantageous platform for inducing tailored metal–support interactions and constructing advanced interfacial structures for multifunctional catalysis.^[^ ^87^ ^]^ Zhuang et al.^[^ ^68^ ^]^ utilized O_v_ in MnFe−MMO as anchoring sites to facilitate the nucleation of high−valence iridium single−metal clusters (Ir−SMCs) via a vacancy−induced mechanism. These Ir−SMCs feature fully exposed catalytic centers and high oxidation potential, enabling efficient oxidation of reduced nicotinamide adenine dinucleotide (NADH). Furthermore, the O_v_ act as bridges for electron transfer within the MnFe−MMO matrix, promoting electron migration from Mn to Fe through the Mn–O_v_–Fe pathway. This process establishes a microelectronic environment conducive to favorable electron states, particularly enhancing the formation of Fe^2+^ species, which are crucial for the Fenton reaction, thereby boosting the generation efficiency of ROS (Figure 3C). This demonstrates the effectiveness of vacancy engineering and topological transformation in creating advanced LDHs−based nanozyme with precisely modulated electronic structures and interfacial properties.

### 2.3 Loading− and Alloyed−type LDHs−Based Nanozyme

Noble metal^[^ ^88^ ^]^ and oxide,^[^ ^89^ ^]^ such as platinum (Pt),^[^ ^90^ ^]^ iridium oxide (IrO_2_),^[^ ^91^ ^]^ and ruthenium oxide (RuO_2_),^[^ ^92^ ^]^ exhibits significant potential for generating ROS. However, the high cost and scarcity hinder the widespread application.^[^ ^93^ ^]^ Therefore, reducing the usage or optimizing the atomic utilization efficiency of precious metal is crucial for advancing the development of low−cost LDHs−based nanozyme. An effective strategy involves uniformly dispersing metal elements as atomic clusters on the support surface, for which the layers of LDHs provide an ideal carrier. Supported nanoclusters or alloyed LDHs achieve a dual leap in active site density and atom utilization by anchoring highly active nanoparticles onto their layers. Compared to conventional LDHs, these type LDHs−based nanozyme exhibits extensively enhanced synergistic effects, leading to superior catalytic efficiency and selectivity. The microwave process provides a robust solution for achieving ultra−low loading. Within an extremely short duration, platinum atomic clusters (PtACs, with a particle size of 2.02 nm) can be loaded onto the surface of NiFe−LDHs.^[^ ^94^, ^95^ ^]^ Beyond this, the use of sodium borohydride (NaBH_4_) as a strong reducing agent also constitutes an effective strategy. In particular, certain noble metals readily form alloys with transition metals (such as Cu and Pt^[^ ^96^ ^]^), which establishes a foundation for providing diverse catalytic sites^[^ ^69^ ^]^ (Figure 3D).

### 2.4 High Entropy LDHs−Based Nanozyme

High entropy nanomaterials (HEMs) have recently garnered much attention. HEMs are new single−phase solid solutions that contain five or more metal elements in near−equimolar concentrations within a single sublattice.^[^ ^97^ ^]^ HEMs exhibit a lattice distortion effect caused by the different sizes of the atoms, facilitating the transportation of active species. Additionally, the synergistic effect of multiple elements initiates cascade reactions in vivo and regulates the biological microenvironment. Therefore, the unique structures and fascinating physicochemical properties of HEMs make them an important class in the catalysis and energy fields.^[^ ^98^ ^]^ Wang et al.^[^ ^99^ ^]^ presents a versatile high−entropy 2D LDHs (HE−LDH) nano−platform that leverages diverse physicochemical advantages to reprogram the TME. In response to the TME, HE−LDHs sequentially release Co^2+^, Fe^3+^, and Cu^2+^ metal ions, exhibiting multi−enzyme activities including SOD, POD, and GP_x_−like activity. These catalytic properties convert specific tumor metabolites into a sustained supply of cytotoxic ROS under TME, thereby alleviating hypoxia.

MMOs, derived from the topological transformation of LDHs precursors, is widely utilized due to the advantage of metal homogeneity.^[^ ^100^ ^]^ Thermal treatment at specific temperature provides a facile route to obtain MMOs with multiple unique properties, such as large surface area and porosity, high thermal stability, and excellent dispersion of metal oxides. One of the most intriguing properties achieved through the use of LDHs precursors is the synergistic effect between different metal oxides. Significant attention has been given to the heterogeneous interfaces formed via the topological transformation of LDHs, as these involve a small number of coordinatively unsaturated active sites at edge and corner positions, which contribute to performance enhancement. Notably, high−entropy oxides (HEOs) have recently come into focus, benefiting from the high−entropy effect, lattice distortion effect, sluggish diffusion effect, and cocktail effect.^[^ ^101^ ^]^ Song et al.^[^ ^70^ ^]^ reported the synthesis of HEOs derived from high−entropy LDHs (HE−LDHs) as precursors at various temperatures (Figure 3E). Among these, the material obtained at 300 temperature exhibited the most potent anti−tumor efficacy, attributable to the synergistic effects among various transition metal elements in oxide−hydroxide mixture, analogous to a “nanotripod”. These work exemplifies the great potential of high−entropy LDHs−based nanozyme in creating sophisticated nanozyme systems capable of multi−enzyme cascade reactions for tumor microenvironment regulation.

### 2.5 Organic−Inorganic Composite LDHs−Based Nanozyme

Recent studies indicate that the incorporation of inorganic fillers, particularly those embedded within the composite network, can sensitively modulate the rheological properties of the organic matrix.^[^ ^71^ ^]^ Consequently, organically−inorganic composite LDHs combine the stability and ordered structure of inorganic layers with the flexibility, high reactivity, and functional diversity of organic components, demonstrating synergistically enhanced properties and broad application potential. Specifically, the inorganic framework confers excellent thermal stability and mechanical strength, while organic modification further improves interfacial compatibility and dispersibility. Lee et al.^[^ ^102^ ^]^ prepared organically intercalated LDHs/ethylene vinyl acetate (EVA) nanocomposites via a solution mixing method, employing suitable anionic surfactants for the organic intercalation of LDHs, which substantially enhanced the thermal stability and mechanical properties of the EVA material. Lv et al.^[^ ^103^ ^]^ uniformly attached Mg−Al layered double hydroxide to the inner walls of wood via an in situ hydrothermal method, generating spherical aggregates. This approach effectively inhibited flame propagation and smoke diffusion, while simultaneously enhancing tensile and compressive strength. Ai et al.^[^ ^71^ ^]^ prepared a highly shear−responsive injectable composite double−network hydrogel as a liquid embolic agent by crosslinking polyvinyl alcohol (PVA) and carboxymethyl chitosan via boronic ester and benzamide dynamic covalent bonds (Figure 3F). The incorporation of 2D LDHs into the network through physical interactions obviously reduced the yield stress during hydrogel injection and enabled the loading of therapeutic agents such as indocyanine green and daunorubicin for photothermal therapy and chemotherapy.

Despite the remarkable tunability of LDHs−based nanozyme, their compositional diversity is constrained by the inherent structural limitations of LDHs layers. The LDHs structure primarily accommodates medium−sized transition metals (e.g., Fe, Co, Ni, Mg, Al), while excluding many biologically relevant elements. Large−radius metals (Sn, Pb, Sb) and precious metals (Pt, Ir) demonstrate poor stability within the brucite−like layers due to mismatched ionic radii and incompatible coordination preferences. This restriction narrows the spectrum of accessible catalytic sites and potentially limits the enzymatic functionalities that can be engineered. To overcome this constraint, researchers are developing innovative strategies including surface anchoring of excluded metal complexes, construction of LDHs−heterostructure hybrids, and post−synthetic intercalation techniques. These approaches expand the elemental diversity beyond intrinsic limitations while maintaining the structural advantages of LDHs platforms, thereby opening new pathways to tailor nanozyme activities for specialized therapeutic applications.

## 3 The Design of LDHs−Based Oxidoreductase for Remodeling Redox Homeostasis

The core mechanism by which LDHs−based nanozyme remodel redox homeostasis lies in the ability to mimic natural enzymes activities, enabling precise regulation of ROS and related free radicals in pathological regions such as tumors or tissue injury. This restores redox homeostasis, thereby suppressing excessive oxidative stress or harnessing ROS for therapeutic effects. **Table** **1** has summarized the key aspects related to the modulation of pathological microenvironments through remodeling redox homeostasis.

**Table 1:** The LDHs−based nanozyme materials in key aspects related to the modulation of remodeling redox homeostasis.

Function	Nanozyme	Enzyme−like activity	Therapeutic mechanism	Reference
Upregulating Redox Homeostasis	PdCu_0.8_@LDH	OXD; GP_x_; NO_x_	SDT	[69]
	HEO	POD; OXD; NO_x_	CDT	[70]
	Au−Cu_4_O_3_/re−LDHs	POD; GO_x_; NO_x_	CDT + Immunotherapy	[104]
	G@Ir/MnFe MMO	POD; NO_x_	PTT + CDT + Immunotherapy	[68]
	CuPt Alloy@LDHs	POD; OXD; NO_x_	CDT	[96]
	S2MFL−LDH	POD; OXD	CDT	[66]
	MgCaFe−LDH@Au	POD; NO_x_ GO_x_; GP_x_	SDT	[105]
	LDH−PAA@DOX	POD	CDT + SDT	[106]
	ICG/Fe−LDH@PEG	POD	CDT + PDT	[107]
	MLLN@HA	POD	CDT + Immunotherapy	[108]
	a−LDH@YW3−56−PEG	OXD	SDT + Immunotherapy	[109]
	403@DR−LDH−PEG	OXD	PDT + Immunotherapy	[110]
	HE−LDHs	POD; GP_x_	CDT + Immunotherapy	[99]
	ZCAG NSs	OXD; GP_x_	SDT + Immunotherapy	[111]
	a−CoBiMn−LDH−PEG	OXD; GP_x_	SDT	[112]
	NiFeMnCu−LDH	POD; OXD; GP_x_	CDT	[113]
	BLD	POD; GP_x_	CDT + Immunotherapy	[114]
	GO_x_/MgAl−SO_3_ LDH	GO_x_	Gas therapy	[115]
	NiFe−LDH−PEG	POD; GP_x_	PCT	[116]
	Cu−LDH	POD	Immunotherapy	[116]
	Co−Fe LDHs@Au@MA	POD; OXD; GP_x_	PTT	[117]
	MCF	POD; GP_x_	SDT + Immunotherapy	[47]
	CDs/LDHzyme	POD	CDT	[118]
	MMF−NSs	POD; OXD; GP_x_	SDT + Immunotherapy	[119]
Downregulating Redox Homeostasis	CoAlEu_0.2_−LDH	SOD	Scavenge ROS	[120]
	CUR/ZnCe−LRH	SOD; CAT	Scavenge ROS + Anti−inflammatory	[121]
	AFGd−LDH	SOD	Scavenge ROS + BBB Targeting	[122]
	ALDzyme	SOD; CAT	Scavenge ROS	[123]
	CeMn−zyme	SOD; CAT	Scavenge ROS	[124]
	Res@LDH	SOD	Scavenge ROS + Anti−inflammatory	[125]
	LDH@miR−182	SOD	Scavenge ROS + Inhibition of pyroptosis	[126]
	ULDH	SOD	Scavenge ROS + Neural regeneration	[67]
	m−CoAl−LDH	SOD	Scavenge ROS	[127]
	LDH@TAGel	SOD	Scavenge ROS + Antiapoptosis	[128]
	CMA	SOD; CAT	Scavenge ROS	[129]
	Ce−LDH NPs	SOD	Scavenge ROS	[130]
	dLDHaHtSC	SOD; CAT	Scavenge ROS	[131]
	a−MgAl−LDH	SOD	Scavenge ROS	[132]
	AA@LDH	APX; SOD	Antibacterial + Osteointegration	[133]

### 3.1 Oxidative LDHs−Based Nanozyme

In the design of LDHs−based nanozyme for advanced tumor therapy, the core strategy for efficient ROS generation involves incorporating transition metals with variable valence states, such as Fe, Cu, and Mn, into the layers. These metals trigger Fenton or Fenton−like reactions in the acidic TME, directly converting H_2_O_2_ into highly toxic ROS. Concurrently, photosensitizer molecules intercalated into the LDHs interlayers can be activated by external light to produce singlet oxygen (^1^O_2_). This synergistic combination of chemodynamic (CDT) therapy and photodynamic (PTT) therapy, achieved through valence state modulation and spatially organized assembly of functional components, significantly enhances ROS production and effectively kills tumor cells. In tumor therapy, LDHs−based nanozyme is engineered to mimic multiple nanozyme activities, such as exhibiting POD−, CAT−, or SOD−like activities, either through cargo loading or intrinsic catalytic behavior. These enzymatic activities allow precise regulation of ROS levels within the TME. On one hand, CAT−like activity decomposes H_2_O_2_ to generate O_2_, alleviating tumor hypoxia and suppressing excessive angiogenesis. On the other hand, elevated ROS can induce tumor cell apoptosis and ferroptosis. Furthermore, LDHs−based nanozyme can synergize with combinded therapy to enhance antitumor efficacy while protecting normal tissues by modulating oxidative stress.^[^ ^134^ ^]^

#### 3.1.1 Peroxidase−Like (POD−Like) Activity

As one of the key executors in regulating the redox pathological microenvironment, peroxidase−like nanozyme catalyze substrate oxidation to generate •OH by using the H_2_O_2_ as an electron acceptor for cancer CDT therapy.^[^ ^135^ ^]^ For example, Yang et al.^[^ ^136^ ^]^ inspired by the artemisinin can cure malaria, which was won Nobel prize, have anchored the artemisinin on the LDHs ultrathin nanosheet, denoted as A@P/uLDH. Initially, the Fe^3+^ on the LDHs ultrathin nanosheet can be partially reduced by the GSH into Fe^2+^ with POD−like activity. In addition, the peroxide bridge of artemisinin can be ruptured by Fe^2+^, resulting to the abundant generation of carbon free radicals, further inducing ferroptosis. To further enhance the activity of POD−like enzymes, it can be achieved through external stimulation^[^ ^137^ ^]^ or by initiating a cascade reaction. Inspired by the cholesterol treatment sodium nitroprusside. Guan's team constructed SNP/LDH composite nanozymes through intercalation assembly. Under the stimulation of the TME, the laminates of SNP/LDH is responsive to weak acids, possess POD−like activity, and can generate ROS. SNP/LDHs release metal ions and SNPS simultaneously, activating SNPS in situ with H_2_O_2_ to generate reactive nitrogen species (RNS), which further undergo a cascade reaction with tumor microcells to produce RNOS with stronger oxidizing properties, thereby enhancing their activity.^[^ ^66^ ^]^

**Figure 4:**
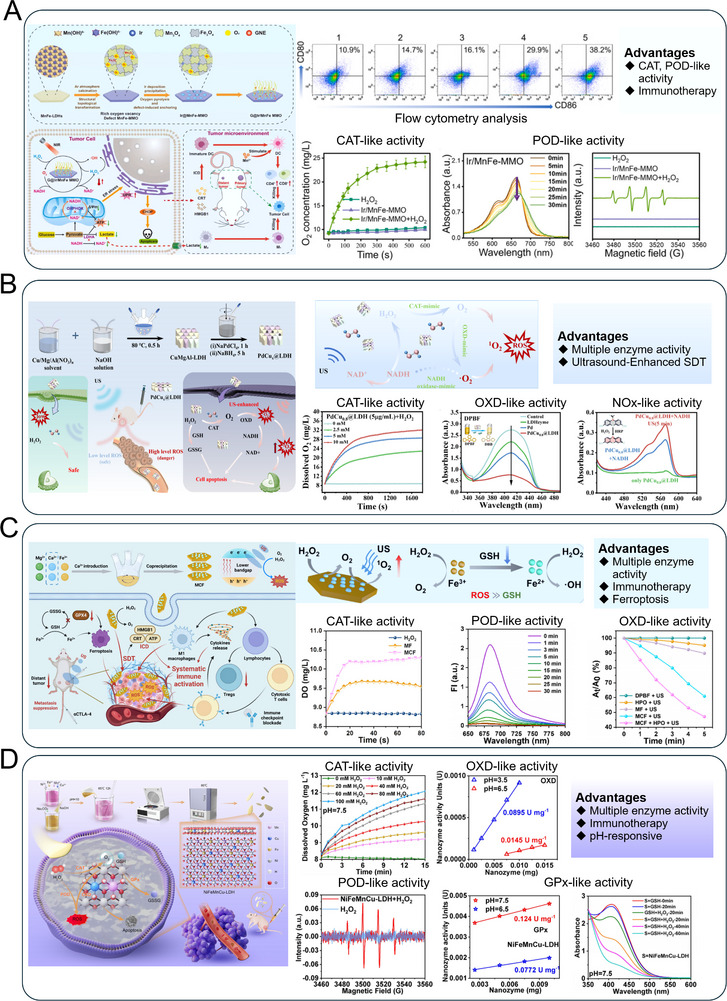
LDHs−based nanozyme upregulate redox homeostasis through enzyme−mimicking reactions for advanced tumor therapy. A) The G@Ir/MnFe MMO obtained through topological transformation and the application in cancer immunotherapy. Reproduced (Adapted) with permission.^[^ ^68^ ^]^ Copyright 2025, Elsevier. B) The PdCu_x_@LDH nanozyme for US−enhanced catalytic and sonodynamic therapy via multienzyme−mimicking cascade reactions and mitochondrial disruption. Reproduced (Adapted) with permission.^[^ ^69^ ^]^ Copyright 2024, American Chemical Society. C) The bioactive MCF−LDH sonosensitizers that integrate TME regulation, SDT, and immunological effects for efficient cancer therapy. Reproduced (Adapted) with permission.^[^ ^47^ ^]^ Copyright 2023, Elsevier. D) The medium−entropy NiFeMnCu−LDH nanozyme for catalytic therapy of primary liver cancer via tumor microenvironment reprogramming. Reproduced (Adapted) with permission.^[^ ^113^ ^]^ Copyright 2025, Wiley.

He's group utilized LDHs as the precursor, have successfully fabricated Au−Cu_4_O_3_/re−LDH nanozyme.^[^ ^104^ ^]^ By virtue of the anchor of Cu_4_O_3_, this ultra small Au site catalases the H_2_O_2_ in TME into •OH, which can enhance the ROS level. In **Figure** **4**A, the nanozyme consists of MnFe mixed metal oxide (MnFe−MMO) nanosheets with abundant O_v_)anchoring ultrasmall Ir−supported metal clusters (Ir−SMCs), forming Mn–O–Fe electron bridges that facilitate directional electron transfer.^[^ ^68^ ^]^ Under 808 nm laser irradiation, it significantly accelerates ROS generation and promotes efficient NADH oxidation, disrupting redox homeostasis and depleting ATP through POD−like activity. This leads to mitochondrial membrane depolarization and sustained endoplasmic reticulum stress, amplifying damage−associated molecular pattern (DAMP) release and robust ICD. Co−loaded with the LDHA inhibitor GNE−140, the system further quenches lactate production and blocks NAD^+^ regeneration, alleviating immunosuppression and enhancing immune cell activity within the TME. Ultimately, G@Ir/MnFe−MMO induces profound immune infiltration and systemic antitumor responses, effectively suppressing both primary and distant tumors.

#### 3.1.2 Oxidase−Like (OXD−Like) Activity

The oxidase−like (OXD−like) activity in tumor therapy represents another advanced enzymatic strategy based on LDHs−based nanozyme. The mechanism relies on the ability of LDHs nanosheets, due to the specific metal ion composition and surface electronic structure, to mimic natural oxidase enzymes under the weakly acidic conditions in TME. This enables the direct catalytic oxidation of intracellular O_2_, accompanied by efficient electron transfer that generates toxic intermediates. Importantly, this process can be synergistically enhanced by catalase (CAT)−like activity, which helps alleviate the limitation of substrate insufficiency.^[^ ^138^ ^]^

Therefore, the generation of intermediates and the electron transfer processes considerably influence the properties of OXD−like activity. Notably, palladium nanocrystals exhibit facet−dependent CAT−like and OXD−like activities. Additionally, Pd has the highest decomposition energy barrier for H_2_O_2_ compared to other noble metals.^[^ ^139^ ^]^ Therefore, by finely regulating the structure and morphology, designing Pd−based nanozymes that simultaneously expose Pd (111) and Pd (100) facets is preferred to achieve synergistic CAT−like and OXD−like dual enzymatic activities, thereby efficiently generating highly toxic and long−lived ^1^O_2_ through cascade catalytic reactions. Guan's group have fabricated a series of LDHs−based PdCu_x_@LDH alloy nanozymes with metal molar ratios (*x* = 0.4 to 1.2) were synthesized via an impregnation−reduction method^[^ ^69^ ^]^ (Figure 4B). These nanozymes can simultaneously expose two active centers of Pd (111) and Pd (100) facets, exhibiting both CAT− and OXD−like activities. Among PdCu_x_@LDH, the PdCu_0.8_@LDH alloy nanozyme shows the highest ROS generation performance due to the synergistic electron transfer effect between Pd and Cu. Under ultrasound (US) stimulation, the multi−enzymatic activity of PdCu_0.8_@LDH can be further enhanced, demonstrating excellent ROS generation activity ≈9 times higher than commercial TiO_2_. In addition, the OXD−like activity can also consume NADH/ lactic acid, inhibits tumor metabolism.

Constructed through the supramolecular intercalation of sodium nitroprusside (SNP) into MgMnFe−layered double hydroxide (2MFL), the S2MFL nanozyme exhibits POD−, CAT−, and OXD−like activities under TME stimulation.^[^ ^66^ ^]^ It reveals remarkable OXD−like activity to generates •O_2_^−^, and reacts with GSH−responsive NO release to produce RNS. The synergistic effect of ROS and RNS disrupts intratumoral redox homeostasis and potently inhibits cancer cells growth without external energy input. In addition, Wang et al.^[^ ^105^ ^]^ develops a multifunctional sonosensitizer based on Ca^2+^−doped MgCaFe−LDH to enhance sonodynamic therapy (SDT) and immunotherapy (Figure 4C). The incorporation of Ca^2+^ optimizes the electronic structure and bandgap, significantly improving SDT efficiency. Additionally, the material modulates TME by alleviating hypoxia and reducing glutathione levels, thereby enhancing oxidative stress. The released Mg^2+^ and Ca^2+^ ions synergistically activate and promote the infiltration of CD8^+^ and CD4^+^ T cells. When combined with anti−CTLA−4 immunotherapy, the system effectively suppresses tumor growth in bilateral models, demonstrating a powerful strategy for integrating SDT, TME regulation, and immune activation.

#### 3.1.3 Glutathione Peroxidase−like (GP_x_−Like) Activity

**Figure 5:**
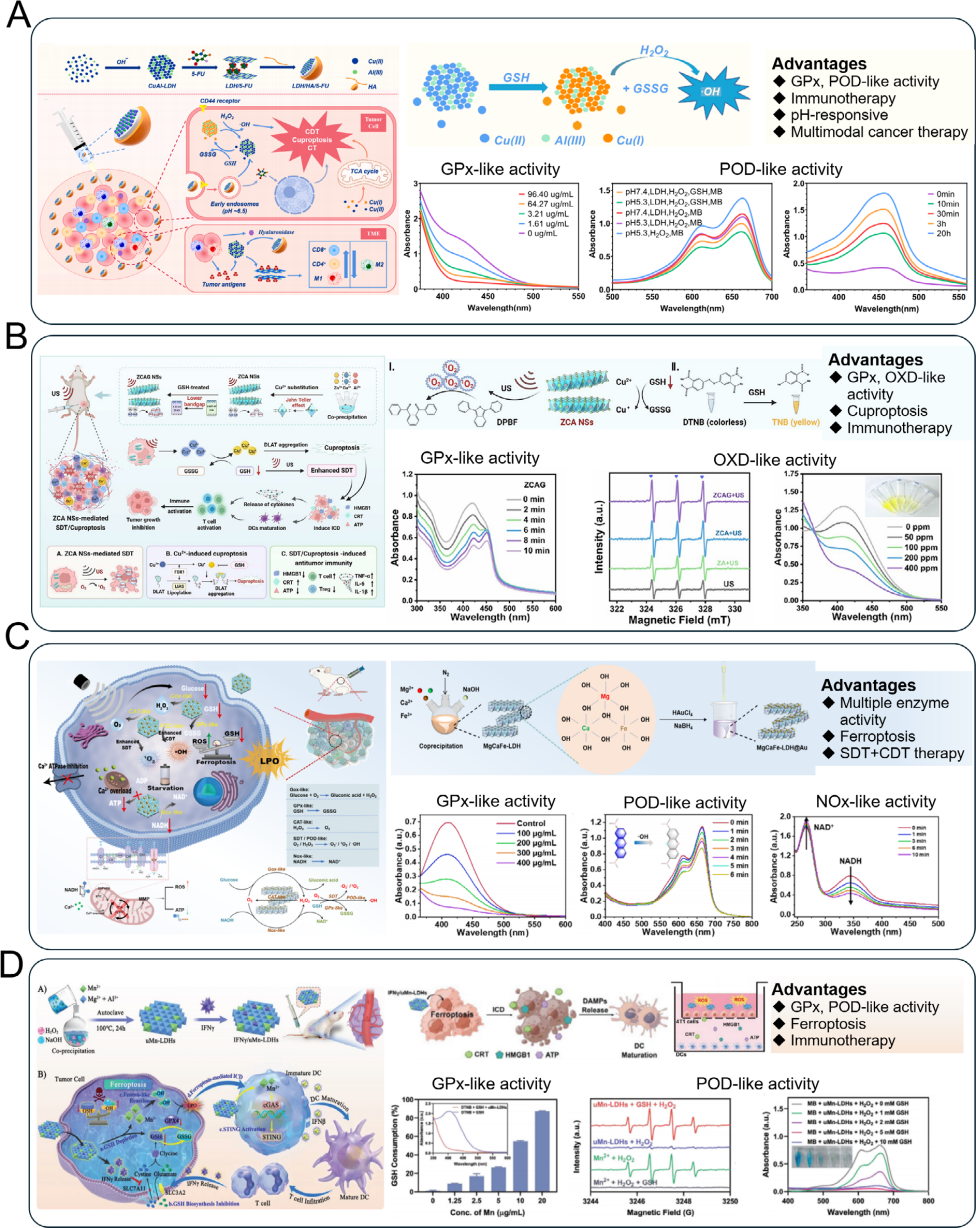
LDHs−based nanozyme upregulate redox homeostasis through enzyme−mimicking reactions for advanced tumor therapy. A) A multifunctional LDH/HA/5−FU for synergistic chemotherapy, chemodynamic therapy, cuproptosis, and immunotherapy. Reproduced (Adapted) with permission.^[^ ^142^ ^]^ Copyright 2023, Elsevier. B) The Cu−substituted ZnAl−LDHs nanosheets (ZCANSs) for synergistic sonodynamic/ cuproptosis therapy and antitumor immunity activation. Reproduced (Adapted) with permission.^[^ ^111^ ^]^ Copyright 2024, American Chemical Society. C) The multi−enzyme mimicking nanozyme MgCaFe−LDH@Au NSs for synergistic catalytic and sonodynamic therapy via TME−specific redox dysregulation and metabolic interference. Reproduced (Adapted) with permission.^[^ ^105^ ^]^ Copyright 2025, American Chemical Society. D) The IFN−γ/uMn−LDH nanosystem for closed−loop ferroptosis−immunotherapy via mutual regulation of redox homeostasis and antitumor immunity. Reproduced (Adapted) with permission.^[^ ^143^ ^]^ Copyright 2023, Wiley.

Glutathione peroxidase (GP_x_), has been well−known for its ability to catalyze the reduction of H_2_O_2_/organic hydroperoxides to H_2_O/alcohols under reduced glutathione (GSH).^[^ ^140^ ^]^ In the TME, glutathione is no longer merely an antioxidant guardian. On the contrary, its function is adeptly exploited by cancer cells, transforming it into an accomplice that promotes tumor survival, progression, and therapy resistance. This central role is achieved by maintaining redox homeostasis within cancer cells through scavenging excess ROS generated by nanozymes. Specifically, moderate levels of ROS can promote cancer cell proliferation and signaling, whereas excessive ROS induces cell death.^[^ ^141^ ^]^ Therefore, cancer cells must activate a robust antioxidant system to keep ROS levels within a “beneficial yet controllable” range. For example, PdCu_0.8_@LDH efficiently decomposes overexpressed GSH via the redox reaction (Cu^2+^ + GSH → Cu^+^ + GSSG), generating oxidized glutathione (GSSG) to avoid non−therapeutic consumption of ROS. Xu et al.^[^ ^113^ ^]^ developed a tetra−component medium−entropy LDHs−based nanozyme, designated as NiFeMnCu−LDH (Figure 4D). In vitro assays demonstrated that efficient ROS generation and GSH depletion induced significant apoptosis in tumor cells, providing a framework for the precise engineering of nanozymes in advanced hepatocellular carcinoma therapy. Xia et al.^[^ ^142^ ^]^ conceived and fabricated a novel organic−inorganic hybrid drug delivery system (LDH/HA/5−FU) by intercalating 5−fluorouracil (5−FU) into the interlayer of CuAl−LDH via an ion−exchange strategy, while adsorbing hyaluronic acid (HA) onto its surface (**Figure** **5**A). This system enables the sustained intracellular depletion of GSH and generation of toxic •OH through a catalytic cycle involving Cu^+^/Cu^2+^ redox reactions. ZCANSs function as both sonosensitizers and copper delivery agents. The incorporation of Cu^2+^ induces a Jahn–Teller effect, optimizing the electronic structure to enhance ROS generation under US irradiation (Figure 5B). Simultaneously, ZCANSs deplete GSH to amplify oxidative stress and remodel the tumor microenvironment. The released Cu^2+^ ions induce proteotoxic stress and trigger cuproptosis, which is further enhanced by sonodynamic therapy. This combination promotes ICD and dendritic cell (DC) maturation in vitro, and effectively eradicates primary tumors while inhibiting lung and liver metastasis by activating robust systemic antitumor immunity in vivo.

#### 3.1.4 Glutathione Oxidase−Like (GSHOx −Like) Activity

The underlying mechanism involves a two−step cascade reaction. First, the surface active sites catalyze the reduction of peroxides into harmless products. Subsequently, the oxidized nanozyme is regenerated by acquiring electrons from reduced glutathione (GSH), simultaneously generating GSSG.^[^ ^144^ ^]^ The ingenuity of this principle lies in its dual−effect precise modulation of cellular redox balance: it simultaneously eliminates the source of oxidative stress and depletes the key intracellular antioxidant, GSH. This GSHOx−like activity is critical in biotherapy. For antibacterial applications, the LDHs−based nanozyme catalytically consume intracellular GSH in bacteria, disrupting their antioxidant defense system and rendering them more susceptible to oxidative killing.^[^ ^145^ ^]^ In cancer therapy, it can specifically deplete GSH within the TME. This action not only directly disrupts the redox balance of tumor cells but also reverses GSH−mediated chemoresistance, thereby significantly enhancing anticancer efficacy and demonstrating substantial application potential.

Zhu et al.^[^ ^146^ ^]^ constructed a multi−enzyme mimicking nanomaterial based on copper−doped cobalt−iron LDHs (CoFeCu−LDH), in which the GSHOx−like activity played a pivotal role in antibacterial therapy. Their study revealed that the introduction of Cu atom can significantly enhance the GSHOx−like activity of the material, enabling efficient depletion of the highly concentrated GSH in the bacterial microenvironment and thereby weakening its antioxidant defense capacity. Under a combined low concentration of H_2_O_2_, CoFeCu−LDH, by synergistically exploiting its POD−like and GSHOx−like activities, substantially increased the accumulation of ROS. This led to bacterial membrane damage and metabolic disruption, resulting in potent bactericidal effects against both Staphylococcus aureus and Escherichia coli. This strategy provides a promising approach for enzyme−catalytic antibacterial therapy based on GSH depletion.

#### 3.1.5 Nicotinamide Adenine Dinucleotide Oxidase−Like (NO_x_−Like) Activity

The primary function of nicotinamide adenine dinucleotide (NADH) oxidase is catalyze the NADH to NAD^+^, typically using molecular O_2_ as the terminal electron acceptor, thereby directly generating • O_2_^−^ or H_2_O_2_.^[^ ^147^ ^]^ As a result, it serves as a significant source of ROS within pathological microenvironments. The canonical reaction catalyzed by NADH oxidase is NADH + H^+^ + O_2_ → NAD^+^ + H_2_O_2_, although • O_2_^−^ may also be produced alternatively. This reaction plays a crucial role in modulating the redox microenvironment. On one hand, it directly produces ROS, contributing to an oxidative stress milieu. Under pathological conditions, aberrant upregulation of this activity leads to sustained accumulation of ROS, triggering oxidative stress that damages DNA, proteins, and lipids, thereby promoting inflammation and apoptosis. On the other hand, it depletes NADH, a central reducing equivalent essential for cellular energy metabolism and antioxidant defense.^[^ ^148^ ^]^ Consequently, excessive NADH consumption disrupts cellular energy metabolism and diminishes antioxidant capacity, rendering cells more vulnerable to existing oxidative challenges and establishing a vicious cycle that ultimately drives cell death. Zheng et al.^[^ ^105^ ^]^ engineered a 2D LDHs−based nanozyme loaded with Au nanoparticles and co−incorporated with bioactive Ca^2+^ and Fe^3+^ ions (denoted as MgCaFe−LDH@Au NSs), tailored to address the specific demands of the TME (Figure 5C). It mimics NO_x_−like activity, impairing the NADH/NAD⁺ redox balance. This compromises the proton motive force necessary for lactate production from pyruvate in aerobic glycolysis and disrupts the mitochondrial electron transport chain involved in oxidative phosphorylation, thereby inhibiting ATP synthesis.

#### 3.1.6 Glucose Oxidase−Like (GO_x_−Like) Activity

Glucose Oxidase (GO_x_), which catalyzes the glucose into glucuronic acid and H_2_O_2_. The core catalytic reaction is β−D−glucose + O_2_ + H_2_O → gluconic acid + H_2_O_2_.^[^ ^149^ ^]^ By persistently consuming ambient glucose and generating gluconic acid which cannot be directly utilized by cells, GO_x_ effectively disrupts the energy supply of tumor. The generated H_2_O_2_ also makes up to the substrate concentration of TME, which recycles the generation of ROS.^[^ ^150^ ^]^ Unlike enzyme activities that primarily function to regulate or scavenge, GO_x_ operates through a more direct mechanism, which actively and continuously depletes nutrients in the pathological microenvironment and converts it into cytotoxic substances, thereby fundamentally reshaping the entire microenvironment.^[^ ^151^ ^]^ He's group^[^ ^104^ ^]^ has fabricated the Au−Cu_4_O_3_/re−LDH. The nanocomposite consists of ultrasmall Au and Cu_4_O_3_ active moieties anchored on reconstructed LDHs nanosheets, forming collaborative redox−active sites with multi−enzyme mimicking activities. It is ultrasmall Au that triggers cascade catalytic reactions that deplete glucose and NADH while generating abundant •OH, leading to severe disruption of mitochondrial electron transport and energy metabolism in 4T1 cells. This metabolic interference not only induces tumor cell apoptosis but also sharply reduces lactate production, thereby alleviating immunosuppression in TME. Ultimately, the nanozyme reprograms the metabolic and immune, robustly enhancing antitumor immunity and effectively inhibiting breast tumor growth and metastasis.

Liu et al.^[^ ^143^ ^]^ proposed a ultrathin manganese−based LDHs (uMn−LDH) nanosheets, which synthesized via a facile solvent−free method and loaded with interferon gamma (IFN−γ) to form IFN−γ/uMn−LDH (Figure 5D). The manganese ions deplete intracellular GSH and promote •OH generation, while released IFN−γ downregulates SLC7A11, further amplifying ferroptosis. This process triggers ICD, which promotes dendritic cell maturation and T cell priming combined with the immunomodulatory function of IFN−γ. Secretion of IFN−γ from activated CD8⁺ T cells in turn reinforces ferroptosis, establishing a self−sustaining antitumor feedback loop. The platform significantly inhibits both primary and distant tumor growth, demonstrating a potent abscopal effect and offering a robust strategy for synergistic ferroptosis and cancer immunotherapy.

### 3.2 Reductive LDHs−Based Nanozyme

When LDHs−based nanozyme is applied to tissue injury repair, the design focus shifts toward scavenging excess ROS to alleviate oxidative stress by leveraging specific valence−changing elements. A key approach involves introducing elements with antioxidant enzyme−mimicking activities. For instance, the Ce^3+^/Ce^4+^ redox pair in cerium−based LDHs mimics catalase and superoxide dismutase, efficiently converting harmful •O_2_^−^ and H_2_O_2_ into harmless O_2_ and H_2_O. Similarly, the Mn^2+^/Mn^3+^ pair in manganese−based LDHs can emulate multiple antioxidant enzymes. By integrating these antioxidant metal elements into the LDHs layers and co−intercalating anions with synergistic antioxidant effects, efficient ROS−scavenging materials can be constructed, creating a favorable microenvironment for tissue regeneration.

On the contrary, in the context of IS treatment, IRI leads to a burst of free radicals, resulting in severe oxidative stress and neuroinflammation.^[^ ^152^ ^]^ IS, accounting for ≈80% of all strokes, cause both disability and death, becoming a huge burden on modern societies. The best method for IS treatment is early vascular recanalization through thrombolysis to restore cerebral blood flow and protect brain tissue. Unfortunately, after the restoration of blood supply to brain, tissue damage is followed, which stands for IRI. This is caused by the excessive generation of ROS in the ischemic penumbra, such as •O_2_^−^, •OH, and H_2_O_2_. Leveraging the efficient SOD− and CAT−like activities, LDHs−based nanozyme effectively scavenge •O_2_^−^ and H_2_O_2_, mitigating oxidative damage and blood−brain barrier disruption. Moreover, the layered structure of LDHs allows intercalation of metal ions such as Mg^2+^ and Zn^2+^, which possess anti−inflammatory and neuroprotective properties.^[^ ^153^ ^]^ Local release of these ions further activates endogenous antioxidant pathways, such as Nrf2, promoting a shift in the microenvironment from pro−inflammatory to pro−regenerative and supporting neural functional recovery. Therefore, owing to the designable nanozyme activities, precise drug/ion delivery capability, and favorable biosafety profiles, LDHs−based nanozyme represent a versatile and efficient nanoplatform for targeted regulation of redox microenvironment, holding significant translational potential.

#### 3.2.1 Catalase−Like (CAT−Like) Activity

**Figure 6:**
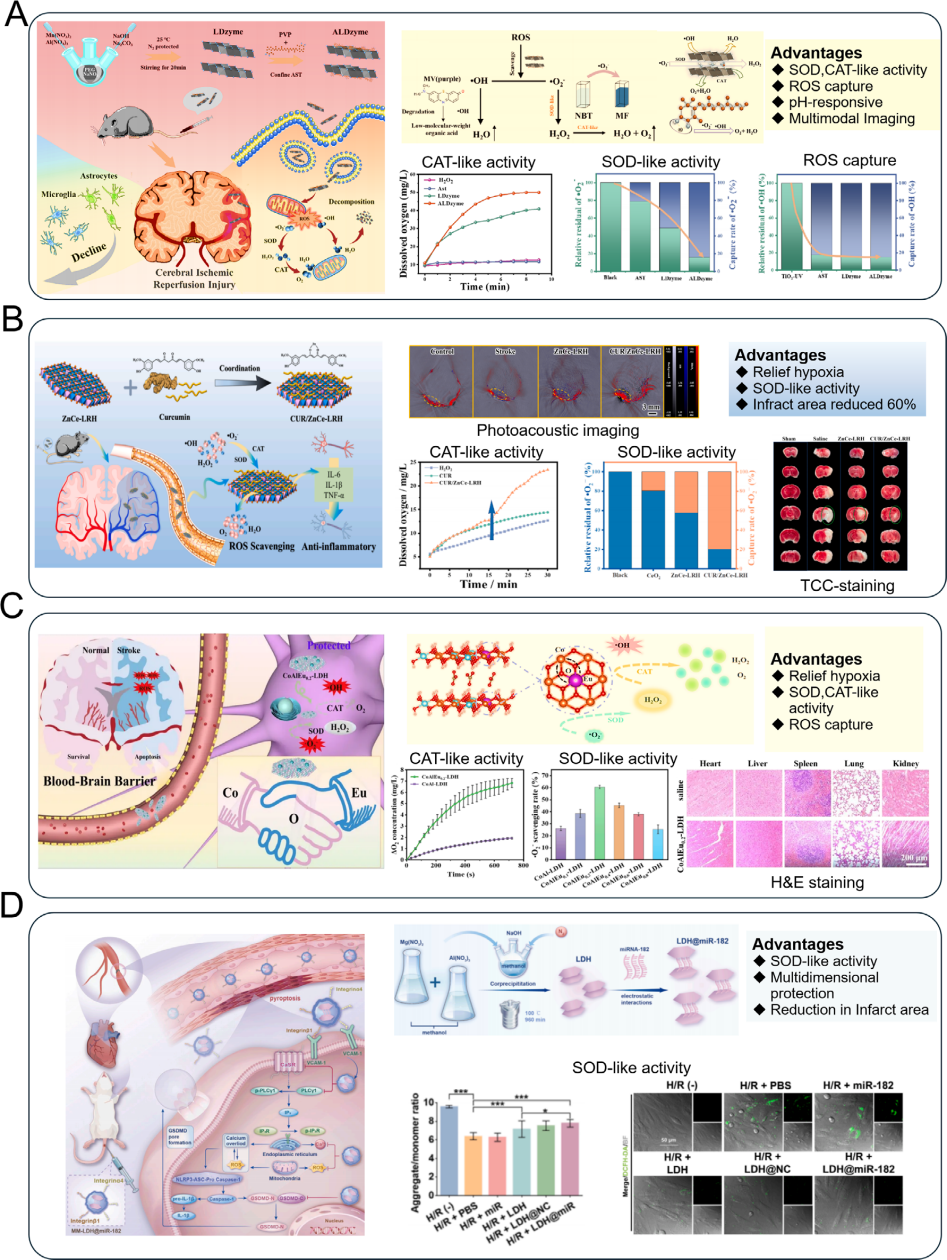
LDHs−based nanozyme downregulate redox homeostasis through enzyme−mimicking reactions for tissue injury. A) ALDzyme modulates the pathological microenvironment through efficiently scavenges ROS, thereby ameliorating the oxidative stress. Reproduced (Adapted) with permission.^[^ ^123^ ^]^ Copyright 2023, American Chemical Society. B) The triple−enzyme mimicking CUR/ZnCe−LRH nanozyme for synergistic anti−oxidative and anti−inflammatory therapy. Reproduced (Adapted) with permission.^[^ ^121^ ^]^ Copyright 2022, Elsevier. C) CoAlEu_0.2_−LDH nanozyme enhances ROS scavenging capacity by facilitating gradient electron transfer through the valence state transition of the rare−earth elements. Reproduced (Adapted) with permission.^[^ ^120^ ^]^ Copyright 2025, Elsevier. D) MM−LDH@miR−182 scavenges ROS and inhibits pyroptosis to ameliorate myocardial ischemia reperfusion injury. Reproduced (Adapted) with permission.^[^ ^126^ ^]^ Copyright 2025, Elsevier.

Catalase (CAT)−like plays an important role in the complex pathophysiology of tissue IRI, serving as a critical defense line against oxidative stress. Although reperfusion restores oxygen and blood supply, it also triggers an oxidative burst, leading to the rapid and massive accumulation of ROS such as •O_2_^−^ and H_2_O_2_. The primary function of CAT−like activity is to efficiently and specifically catalyze the decomposition of H_2_O_2_ into harmless H_2_O and O_2_, thereby directly eliminating this key mediator of oxidative stress.^[^ ^154^ ^]^ If CAT−like activity is insufficient or inhibited, H_2_O_2_ accumulates rapidly and can generate highly destructive •OH via the Fenton reaction. This process initiates a vicious cycle: it attacks cell membranes to cause lipid peroxidation, thereby impairing mitochondrial function and exacerbating the energy crisis, which in turn activates inflammatory pathways and apoptosis. In the brain, this directly damages neurons and compromises the integrity of the blood−brain barrier (BBB). Qu and co−authors^[^ ^155^ ^]^ believe that with the worsening of ischemia, no matter due to prolonged occlusion time or more serious embolism in cerebral arteries, the pathophysiological basis for the failure of interventional thrombectomy or thrombolysis is the unsuccessful oxygen resupply to the distal ischemic tissue, e.g. the ischemic penumbra, after the remove of thrombus from the large artery. Therefore, CAT−like activity might provide a key route to speed up the process for improving the chemical environment in brain to rescue more salvageable neurons in larger areas. Therefore, the level of CAT−like activity directly determines the tissue's capacity to clear H_2_O_2_, representing a critical threshold that limits the expansion of oxidative damage. Maintaining or enhancing CAT−like activity is of universal importance for mitigating reperfusion injury in various organs (e.g., heart, brain, liver, kidney) and preserving tissue function, making it a significant therapeutic target. According to this, Our group have successfully fabricated multiple LDHs−based nanozyme, including MgAlGd/Ferrin/Ato, CUR/ZnCe−LRH, and ALDzyme, respectively^[^ ^121^ ^]^ (**Figure** **6**A). Initially, to pass the BBB, MgAlGd−LDH nanozyme is loaded with Ferrin to increase the accumulation of MgAlGd/Ferrin/Ato and target the lesion. The in vitro confocal imaging experiments reveal the satisfactory CAT−like activity, while in vivo TCC−stained results demonstrate the obvious decreased infract area and declined neurological score. To not only capture ROS, but also ease the hypoxia condition, her group utilize curcumin regarded as enzyme exciter, to construct the CUR/ZnCe−LRH nanozyme.^[^ ^121^ ^]^ This CUR/ZnCe−LRH nanozyme not only possessed of SOD−like activity, but also render CAT−like activity. As revealed in Figure 6B, photoacoustic imaging demonstrates the obvious elevated O_2_ concentration after the administration of CUR/ZnCe−LRH with the MCAO model. This CUR/ZnCe−LRH nanozyme decreases infract area and declined neurological score. Similar result also is found in the ALDzyme work.^[^ ^123^ ^]^

#### 3.2.2 Superoxide Dismutase−Like (SOD−Like) Activity

SOD−like activity can effectively convert toxic ROS (^1^O_2_ and •O_2_^−^) into H_2_O_2_ and O_2_ via dismutation reaction, to ease the reperfusion injury.^[^ ^156^ ^]^ To overcome these barriers, Song et al.^[^ ^120^ ^]^ designed Eu−doped CoAl−LDHs (CoAlEu_0.2_−LDHs) featuring asymmetric transition metal−oxygen−rare earth active site (Figure 6C). This site enables gradient electron migration via orbital hybridization, which not only stabilizes the LDHs framework but also sustains high catalytic activity through rare−earth valence cycling. Experimental and theoretical approaches demonstrate that the Co−O−Eu site facilitates efficient charge transfer, markedly improving ROS scavenging and anti−inflammatory performance, finally overcome IRI. This study provides unconventional insights into the design of advanced rare−earth−doped antioxidant materials, while bridging the gap in therapeutic strategies for cerebral IRI.

For example, Wu et al.^[^ ^126^ ^]^ develops a biomimetic nano−bioplatform (MM−LDH@miR−182) using macrophage membrane−coated LDHs for targeted treatment of myocardial ischemia–reperfusion injury, indicated in Figure 6D. The LDHs component demonstrates intrinsic ROS scavenging ability, which helps reduce oxidative stress and suppress mitochondrial ROS production. Combined with calcium overload regulation via the CaSR/PLCγ1/IP3R pathway, this ROS scavenging effectively inhibits cardiomyocyte pyroptosis, preserves mitochondrial membrane potential, and improves cardiac function. The platform highlights the therapeutic potential of LDHs−based nanomaterials in alleviating oxidative damage and inflammation during ischemic heart injury.

**Figure 7:**
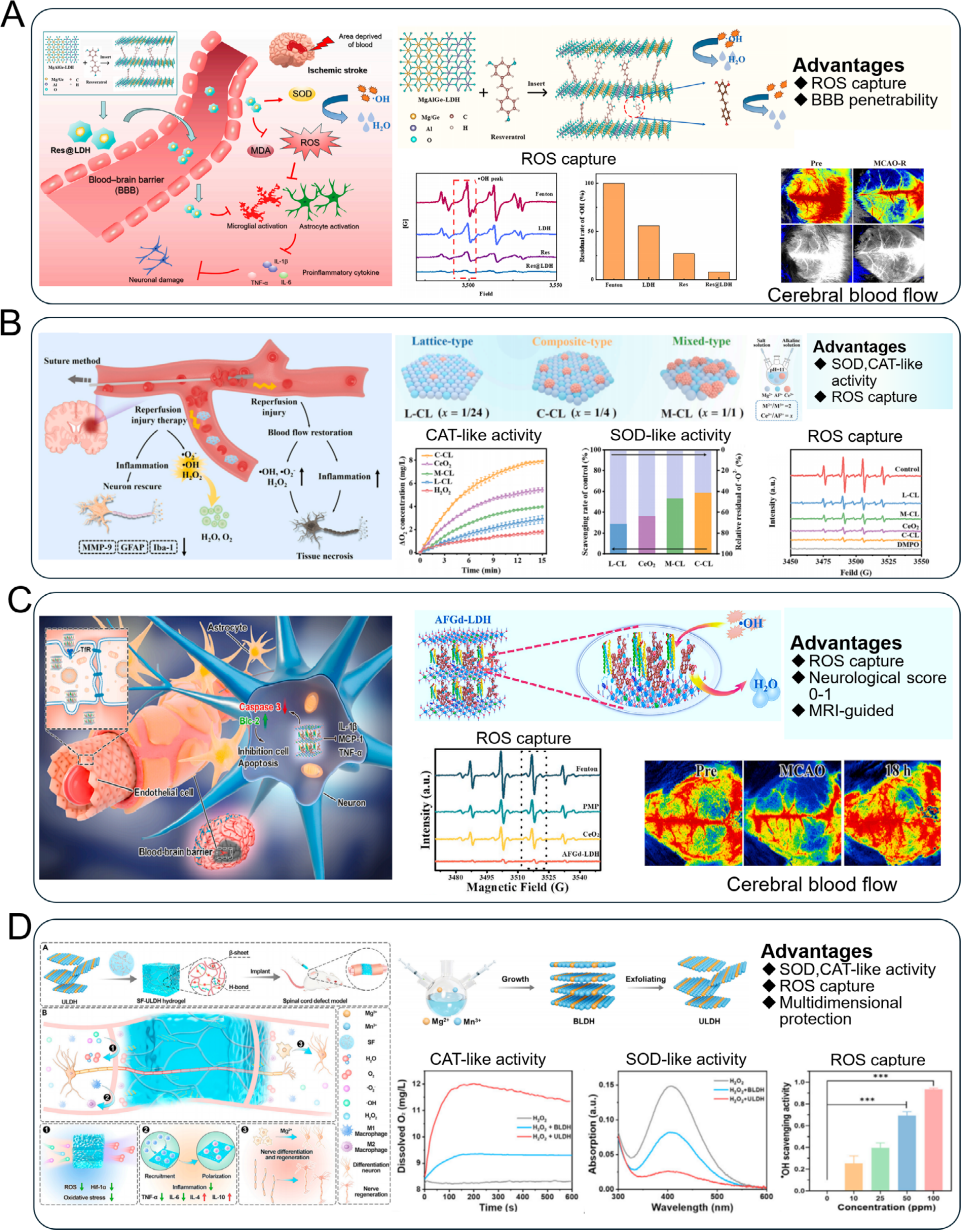
LDHs−based nanozyme downregulate redox homeostasis via enzyme−mimicking reactions for tissue injury. A) The neuroprotective mechanism of Res@LDH nanozyme for ischemic stroke treatment. Reproduced (Adapted) with permission.^[^ ^125^ ^]^ Copyright 2024, Springer Nature. B) C−CL nanozyme with enhanced multi−enzyme mimetic activity scavenges ROS and mitigates cerebral IRI. Reproduced (Adapted) with permission.^[^ ^157^ ^]^ Copyright 2023, Elsevier. C) SF−ULDH hydrogel scavenges ROS, releases O_2_ and Mg^2+^, and promotes neuronal repair in spinal cord injury. Reproduced (Adapted) with permission.^[^ ^67^ ^]^ Copyright 2023, Elsevier. D) The multifunctional neuroprotective nanoplatform AFGd−LDH for imaging−guided therapy of AIS. Reproduced (Adapted) with permission.^[^ ^122^ ^]^ Copyright 2022, Elsevier.

In addition, the Res@LDH nanotherapeutic agent, composed of germanium−containing layered double hydroxide (Ge−LDH) nanosheets loaded with resveratrol, demonstrates enhanced BBB penetration and superior ROS scavenging capacity^[^ ^125^ ^]^ (**Figure** **7**A). It effectively attenuates cerebral ischemia–reperfusion injury by reducing infarct volume, improving neurological function, preserving BBB integrity, suppressing neuroinflammation via inhibition of pro−inflammatory cytokine release, and dampening activation of microglia and astrocytes. Through the potent antioxidative and anti−inflammatory effects, Res@LDH offers a promising therapeutic strategy against IS. Chen and co−authors develop a composite CeO_2_@LDH (C−CL) material that enhances the ROS scavenging capacity of CeO_2_ through electronic and structural modulation via 2D LDHs^[^ ^157^ ^]^ (Figure 7B). The C−CL exhibits significantly improved SOD− and CAT−like activities, efficiently decomposing H_2_O_2_ and scavenging •OH. In a mouse model of IS, C−CL markedly reduces the cerebral infarct area and improves neurological scores, demonstrating great potential for attenuating ischemia/reperfusion injury by mitigating oxidative damage. Wang et al.^[^ ^67^ ^]^ develops an injectable silk fibroin hydrogel integrated with ultrathin MgMn−LDHs (SF−ULDH) for spinal cord injury repair (Figure 7C). The ULDH component continuously scavenges ROS and releases O_2_ to alleviate oxidative stress and hypoxia in the injured microenvironment. In addition, it supplies bioactive Mg^2+^ to promote neuronal cell growth and differentiation. Through multi−pathway regulation including PI3K−Akt and axon regeneration signaling, the SF−ULDH hydrogel effectively improves the pathological microenvironment and enhances functional recovery.

#### 3.2.3 Ascorbate Peroxidase−Like (APX−Like) Activity

Ascorbate peroxidase (APX)−like, primarily found in plants, utilizes ascorbate (vitamin C) to specifically reduce H_2_O_2_ to H_2_O, thereby eliminating ROS and protecting cellular structures. Conversely, while direct APX enzyme activity is insufficient, the underlying antioxidant principle of vitamin C remains identical and crucial. In animal tissue repair, vitamin C acts as a core antioxidant, directly scavenging excess ROS at injury sites to mitigate oxidative stress.^[^ ^158^ ^]^ Simultaneously, functioning as an essential cofactor for collagen synthesis, it directly promotes wound healing, soft tissue repair, and bone regeneration, thus playing a critical role in the repair process. To address the dual challenge of bacterial infection and impaired bone regeneration in orthopedics, a nanozyme−mimicking therapeutic (AA@LDH) is developed by integrating ascorbic acid with Mg–Fe LDHs.^[^ ^133^ ^]^ In the acidic infectious microenvironment, AA@LDH degrades and triggers a pro−oxidative shift in ascorbic acid, generating abundant ROS that disrupts critical bacterial processes, achieving nearly 100% antibacterial efficacy. Simultaneously, the system directly promotes osteogenic differentiation, enhancing ALP activity and matrix mineralization. By concurrently eradicating bacteria and favorably modulating the osteogenic immune microenvironment, AA@LDH demonstrates a synergistic combined therapy, offering a promising strategy for treating bone infections and supporting regeneration.

#### 3.2.4 Glutathione Reductase −Like (GR−Like) Activity

Glutathione reductase (GR)−like is another key enzyme responsible for maintaining intracellular redox homeostasis. Its core catalytic mechanism involves utilizing NADPH as an electron donor to efficiently reduce GSSG to GSH.^[^ ^159^ ^]^ This reaction not only regenerates GSH, the primary intracellular antioxidant, but also sustains the physiological GSH/GSSG ratio, thereby ensuring the continuous operation of the antioxidant defense system.

During animal tissue repair, trauma or inflammation sites generate excessive ROS. By regenerating GSH, GR indirectly supports the GPx−like enzyme activity, enabling the collective clearance of excess ROS and protecting nascent cells from oxidative damage.^[^ ^160^ ^]^ Furthermore, adequate GSH levels are vital for cell proliferation, immune regulation, and the synthesis and cross−linking of collagen. Consequently, GR−like activity directly influences the progression of critical regenerative processes such as wound healing and bone repair, with its functional deficiency potentially delaying tissue regeneration.

It is worth noting that LDHs−based nanozyme offer distinct advantages over conventional nanozyme systems, such as Fe_3_O_4_ or CeO_2_, by combining programmable architecture with multi enzyme mimicry. Unlike traditional nanozymes, which often possess static active sites and limited tunability, LDHs−based nanozyme enable rational design of catalytic properties through layered structure flexibility and diverse host guest chemistry. This programmability allows for the creation of nanozymes with tailored multi enzyme activities, including simultaneous SOD−, CAT−, and POD−like functions, that can be adaptively regulated in response to specific pathological microenvironments. For example, the interlayer space of LDHs−based nanozyme can accommodate therapeutic anions or signaling molecules, enabling synergistic ROS scavenging and drug release. This represents a feature unattainable with monometallic oxide nanozymes. Moreover, the biocompatibility and degradation behavior of LDHs further enhance their suitability for biomedical applications. By moving beyond empirical optimization, LDH based platforms represent a paradigm shift toward predictive nanozyme design, addressing complex redox imbalances with spatial and temporal precision.

### 3.3 Combination Therapy and Integrated Diagnosis Mediated by LDHs−Based Nanozyme

Unlike natural enzymes that typically catalyze a single reaction, LDHs−based nanozyme usually exhibits a distinct advantage in multifunctional enzymatic activity. Due to the tailorable designs and diverse active sites, a single nanozyme often possesses multiple enzyme−like catalytic capabilities.^[^ ^161^ ^]^ These functions operate not in isolation, but synergistically, enabling efficient cascade reactions or parallel pathways that convert various substrates into different products. Such an intrinsic, integrated multi−enzyme system significantly simplifies catalytic processes and enhances reaction efficiency, representing a core competitive edge of nanozymes over natural counterparts.

Combination therapy based on multienzyme−mimetic activity is an innovative strategy that utilizes nanozyme to mimic natural enzyme functions in synergy with physical approaches such as PTT^[^ ^162^ ^]^ and SDT.^[^ ^163^ ^]^ Its core significance lies in the ability to intelligently respond to endogenous signals within the TME, enabling the in situ generation of toxic species or depletion of protective molecules through catalytic reactions, thereby achieving precise antitumor effects. This multimodal synergistic approach significantly overcomes the limitations of monotherapies, enhances treatment efficacy, reduces required dosages, and mitigates systemic toxicity, representing a promising direction for future precision oncology. At the same time, LDHs−based nanozyme, as exceptional nanocarriers, exhibits significant potential in the field of theranostics. Not only do they enable the highly efficient loading of therapeutic agents, but the inherent structural properties also facilitate modification into multimodal imaging agents, thereby achieving precise localization and visualization.

#### 3.3.1 Advanced Therapeutic Approach Based on LDH−Mimetic Oxidoreductase

CDT is an emerging tumor treatment strategy that utilizes endogenous substances within the tumor microenvironment to initiate localized chemical reactions.^[^ ^164^ ^]^ Its core mechanism involves the use of nanocatalytic materials to mimic enzymatic catalysis at tumor sites, leveraging Fenton and Fenton−like reactions among other catalytic pathways to convert overexpressed H_2_O_2_ into highly toxic ROS, such as •OH. These ROS induce severe oxidative stress, causing irreversible damage to critical biomolecules including lipids, proteins, and DNA, ultimately leading to apoptosis and necrosis. By exploiting distinctive features of the tumor microenvironment, such as acidic pH, elevated H_2_O_2_ levels, and altered antioxidant metabolism, CDT achieves targeted tumor cell elimination with high specificity and minimal systemic toxicity. Si et al.^[^ ^165^ ^]^ develops a probiotic−enhanced CDT platform by integrating Lactobacillus acidophilus with CaO_2_−loaded CoFeCe−LDHs (CaO_2_/LDH@L). The probiotic enables improved tumor targeting via hypoxia tropism and metabolically acidifies the tumor microenvironment to pH below 5.5. This acidity, together with O_2_−derived H_2_O_2_, significantly promotes •OH generation via CoFeCe−LDH, leading to highly efficient tumor ablation with an inhibition rate of 96.4%, which is 2.32 times higher than that of the probiotic−free system. The work demonstrates a potent strategy for remodeling the tumor microenvironment to amplify ROS production and enhance CDT efficacy.

The therapeutic mechanism of PTT is based on the photothermal conversion effect. Under laser irradiation, photothermal agents absorb light energy and convert it into heat through non−radiative relaxation, causing a sharp increase in local temperature.^[^ ^166^ ^]^ The resulting high heat can directly induce irreversible damage to tumor cells, such as protein denaturation and cell membrane rupture, ultimately leading to apoptosis or necrosis. It is important to note, however, that this thermal effect may also trigger the upregulation of heat shock proteins (HSPs), which are stress−induced protective proteins. This response may contribute to thermal tolerance in tumor cells and potentially reduce treatment efficacy. Therefore, inhibiting HSP expression has become an important strategy for enhancing the effectiveness of photothermal therapy. Lin et al.^[^ ^167^ ^]^ develops FeMn−LDHs nanozyme as an effective photothermal nanocarrier for multimodal cancer therapy. FeMn−LDHs exhibits significant photothermal properties, which not only enable PTT but also enhance the efficacy of CDT. Under near−infrared light irradiation, the photothermal effect accelerates Fenton−like reactions, improving therapeutic outcomes. This photothermal capability, combined with its responsive degradation in the TME, positions FeMn−LDHs as a key component in synergistic anticancer strategies.

The core mechanism of SDT involves the use of US waves to penetrate deeply into tissues and activate sonosensitizers that have accumulated preferentially in tumor site. Through mechanisms such as acoustic cavitation, the sonosensitizer molecules are excited from their ground state to an excited state.^[^ ^168^ ^]^ Upon de−excitation, they transfer energy to surrounding molecules such as oxygen, leading to the abundant generation of cytotoxic substances, particularly ROS. These highly reactive agents induce severe oxidative stress damage, disrupting lipid membranes, proteins, and DNA structures within tumor cells, ultimately resulting in selective induction of apoptosis or necrosis. By leveraging the strong tissue−penetrating capability of US, SDT offers a non−invasive and precise treatment modality for deep−seated tumors. Hu et al.^[^ ^169^ ^]^ proposed a strategy to enhance SDT by developing amorphous ultrathin CoW−LDH nanosheets (a−CoW−LDH) via acid−induced crystalline to amorphous phase transformation. The resulting LDHs nanosheet exhibits significantly improved US−triggered ROS generation, ≈17 times higher than that of commercial TiO_2_ sonosensitizers, attributed to defect formation and electronic structure modulation. With polyethylene glycol modification, a−CoW−LDH serves as an efficient sonosensitizer, enabling effective tumor cell death in vitro and complete tumor eradication in vivo under US.

Photodynamic therapy (PDT) is a minimally invasive and precision treatment modality based on photochemical reactions. The core mechanism begins with the administration of a photosensitizer, which selectively accumulates in tumor tissues.^[^ ^170^ ^]^ Subsequent irradiation with light of a specific wavelength excites the photosensitizer molecules to an elevated energy state. In this activated form, the photosensitizer transfers energy to surrounding oxygen molecules, generating highly cytotoxic ROS, notably ^1^O_2_. These reactive intermediates cause irreversible oxidative damage to essential cellular structures, including membranes, proteins, and nucleic acids, while also effectively disrupting the tumor vasculature.^[^ ^171^ ^]^ Through this dual mechanism of direct cytotoxicity and indirect nutrient deprivation, PDT efficiently eradicates tumor tissue. Owing to the minimal retention of photosensitizer in normal tissues and the precise control over light delivery, the therapy induces minimal damage to surrounding healthy structures, achieving highly efficient and targeted treatment. Chen et al.^[^ ^110^ ^]^ develops a multifunctional nanoagent (403@DR−LDH) based on defect−rich CoMo−layered double hydroxide nanosheets loaded with a NETosis inhibitor (YW4−03) for enhanced PDT and immunotherapy. The nanosystem exhibits high ^1^O_2_ generation under 1550 nm laser irradiation, which effectively induces immunogenic cell death. Moreover, it inhibits neutrophil extracellular traps formation by downregulating H3cit expression, thereby alleviating an immunosuppressive barrier. Combined with its ability to promote T−cell activation and dendritic cell maturation, 403@DR−LDH significantly inhibits tumor growth, metastasis, and recurrence, while remodeling the tumor immune microenvironment.

Immunotherapy has become a cornerstone of modern oncology by harnessing the body's immune system to deliver targeted and durable antitumor responses.^[^ ^172^ ^]^ It establishes long−term immunological memory, suppressing cancer recurrence and metastasis, while also eliciting systemic effects such as abscopal regression of untreated tumors.^[^ ^173^ ^]^ This approach can reprogram immunosuppressive TME, turning immunologically silent cold tumors into responsive hot ones and improving immune cell infiltration. Furthermore, immunotherapy acts synergistically with other treatment modalities, including ferroptosis inducers and nanocatalytic agents, to amplify therapeutic outcomes. Although challenges like limited tumor infiltration and immune−related adverse effects remain, integrating immunotherapy with biomaterial−based strategies and nanozymes offers a promising avenue for more precise, effective, and sustainable cancer treatment. Fang et al.^[^ ^174^ ^]^ propose a FeAl−LDH nanoplatform, ehich demonstrates pH−sensitive biodegradation in the acidic tumor microenvironment, thereby releasing Fe^2^⁺ ions that catalytically generate ROS via the Fenton reaction. This process effectively induces ferroptosis in tumor cells, characterized by lipid peroxidation and the release of immunogenic damage−associated molecular patterns (DAMPs). Consequently, these DAMPs promote dendritic cell maturation and subsequently activate cytotoxic T cells, initiating a robust adaptive immune response. Furthermore, the Fe^2+^, driven ROS production simultaneously polarizes tumor−associated macrophages toward the antitumor M1 phenotype, thereby reversing immunosuppression. Ultimately, this combined ferroptosis−immunotherapy strategy synergistically enhances antitumor immunity and significantly suppresses tumor growth in a bilateral breast cancer model.

The emerging modalities introduced above, such as PDT, PTT, CDT, and SDT, each possess the potential to be used independently as precise and minimally invasive monotherapies. However, their greater value lies in their exceptional combinatorial potential. These approaches can not only be combined with conventional radio− or chemotherapy and surgery but also be flexibly integrated with one another to form multimodal synergistic antitumor strategies. For instance, the thermal effects of PTT can enhance the reaction kinetics of CDT,^[^ ^175^ ^]^ while cellular damage induced by SDT or PDT may activate immune responses and synergize with immunotherapy. This intrinsic dual capacity, which combines standalone utility with enhanced synergistic effects, enables significant functional integration and mechanistic complementarity. Such combinations effectively overcome tumor resistance, improve treatment efficacy, and suppress recurrence and metastasis, representing a promising direction for the future of precision cancer therapy. To address the immunosuppressive TME, Liu and co−authors^[^ ^176^ ^]^ are applied weakly alkaline layered double hydroxide nanoparticles (LDHNPs) as a neoadjuvant immunotherapy. LDHNPs persistently neutralized tumoral acidity and inhibited tumor cell autophagy by blocking lysosomal function. This remodeling of the TIME promoted the infiltration and activity of antitumor immune cells, including T cells and macrophages. Furthermore, LDHNPs captured released tumor antigens, enhancing antigen presentation. This multi−faceted approach significantly suppressed the growth of melanoma and colon tumors in vivo, demonstrating that LDHNPs act as a potent immunomodulator to “awaken” host immunity, offering a promising strategy for solid tumor immunotherapy. Chen et al.^[^ ^177^ ^]^ develops a FT−BL@P nano−platform for targeted gene therapy against hepatocellular carcinoma (HCC). The system delivers the tumor suppressor gene CHRDL−1, which activates the Hippo pathway, specifically to HCC cells. FT−BL@P effectively inhibits HCC growth and induces apoptosis in vitro and in vivo. Furthermore, a synergistic antitumor effect is achieved when this gene therapy is combined with JPH203, an L−type amino acid transporter 1 (LAT−1) inhibitor. This combination strategy, leveraging both genetic and pharmacological approaches, demonstrates significant potential as a precision therapy for HCC, offering enhanced efficacy by simultaneously targeting multiple pathways.

To address the limitations of current nanozymes in treating bacterial keratitis, a composite nanozyme, DT−ZnFe−LDH@Cu, was constructed by integrating Cu single−atom nanozymes and aminated dextran onto ZnFe−LDH nanosheets.^[^ ^178^ ^]^ This system exhibits triple−enzyme mimetic activities, enabling multi−type ROS generation for effective bacterial eradication. The dextran coating facilitates deep biofilm penetration. Furthermore, the nanozyme demonstrates a combined therapeutic effect by not only eliminating bacteria but also promoting corneal healing through immunomodulation, specifically by driving macrophage polarization from the pro−inflammatory M1 to the anti−inflammatory M2 phenotype and reducing α−SMA expression to minimize scarring. This synergistic strategy outperforms conventional treatments in a BK rabbit model, showcasing its potential as a comprehensive therapy for ocular infections. In addition, to address the challenging microenvironment of inflammatory maxillofacial defects, an artificial antioxidase was developed using Ru−doped LDHs (Ru−hydroxide).^[^ ^179^ ^]^ This material features hydroxyl−synergistic monoatomic Ru centers that enable efficient, broad−spectrum ROS scavenging by facilitating rapid proton and electron transfer. By effectively neutralizing excessive ROS, Ru−hydroxide sustains stem cell viability and promotes osteogenic differentiation under oxidative stress. Furthermore, it modulates the inflammatory microenvironment, thereby supporting maxillofacial bone regeneration in male mice. This multifunctional nanozyme presents a promising strategy for managing inflammation−associated tissue repair, offering potential applications in regenerative medicine for conditions such as arthritis, diabetic wounds, and bone fractures. Yu et al.^[^ ^180^ ^]^ constructed gadolinium(Gd)−doped LDHs (Gd−LDHs) for IS theranostics. The material retained the characteristic layered structure of LDHs, and its unique alkaline layers and intercalated anions conferred pH−responsive drug release capability, enabling the intelligent release of curcumin (CUR) in the acidic ischemic region. The study demonstrated that Gd_0.1_−LDHs exhibited a high longitudinal relaxivity, making them suitable for magnetic resonance imaging. In an in vitro oxygen−glucose deprivation (OGD) model, the drug−loaded composite Gd_0.1_−LDHs/CUR increased the viability of PC12 cells from 75.3% to 88.3%, indicating significant neuroprotective effects.^[^ ^180^ ^]^ The structural stability and surface properties of LDHs synergistically enhanced their performance as a theranostic platform, offering a novel strategy for the treatment of cerebral ischemia. In addition, Lin et al.^[^ ^181^ ^]^ constructed a NIR−II responsive multifunctional scaffold based on MgFe−LDHs for integrated treatment of osteosarcoma after surgery. The Fe^3+^ in the LDHs structure not only conferred excellent photothermal conversion capability for effective PTT therapy under NIR−II laser irradiation, but also catalyzed the Fenton−like reaction to generate •OH from H_2_O_2_, enhancing CDT. The layered structure and alkaline nature of LDHs facilitated efficient curcumin (Cur) loading and its smart release in the acidic TME, enabling synergistic chemotherapy. Moreover, the degradation products of LDHs, including Mg^2+^ and Fe^3+^, together with the alkaline microenvironment and mild hyperthermia, collaboratively promoted angiogenesis and osteogenic differentiation. This system achieved integrated antitumor and bone regeneration, demonstrating the unique advantages of LDHs in structural tunability and multifunctional integration.

#### 3.3.2 Integrating LDHs−Based Redox Enzymes for Theranostics

##### Magnetic Resonance Imaging (MRI)

MRI relies on contrast agents that alter the relaxation times of water protons in tissues to generate signal contrast.^[^ ^182^ ^]^ There are two main types: longitudinal relaxation time (T_1_)−weighted imaging (positive contrast), which requires paramagnetic metal ions (e.g., Gd^3+^, Mn^2+^) to shorten the T_1_ of water protons, resulting in bright image contrast; and transverse relaxation time (T_2_)−weighted imaging (negative contrast), which employs superparamagnetic materials (e.g., Fe_3_O_4_ nanoparticles) to markedly reduce T_2_, producing dark signal regions.^[^ ^183^ ^]^

To confer MRI functionality, several strategies can be employed. First, metal ion doping can be utilized by directly introducing magnetic ions such as Gd^3+^, Mn^2+^, or Fe^3+^ into the host layers of LDHs−based nanozyme (e.g., replacing Mg^2+^ or Al^3+^), thereby imparting intrinsic MRI contrast capabilities. For instance, MgGd−LDH or MnFe−LDH can serve as highly efficient T_1_ contrast agents.^[^ ^184^ ^]^ Second, interlayer loading offers another effective approach, wherein paramagnetic metal complex anions are intercalated as guest species into the interlayer region of LDHs−based nanozyme.^[^ ^185^ ^]^ Finally, surface modification can be applied to anchor or load superparamagnetic iron oxide nanoparticles (SPIONs) onto LDHs nanosheets, constructing T_2_−weighted contrast agents. Compared to other materials, LDHs−based platforms enable high metal ion loading capacity, mitigate the nephrotoxicity risks associated with small−molecule gadolinium−based contrast agents, and promote accumulation at tumor sites via the enhanced permeability and retention (EPR) effect, greatly improving imaging signal−to−noise ratio and specificity.^[^ ^186^ ^]^ The nanoagent is composed of gadolinium−containing layered double hydroxide (Gd−LDH) nanosheets as a carrier and MRI contrast agent, atorvastatin (ATO) as a neuroprotective drug, and ferritin heavy chain (FTH) to facilitate blood−brain barrier transport (Figure 7D).^[^ ^122^ ^]^ AFGd−LDH exhibits exceptional ROS scavenging efficiency (∼90%), significantly surpassing conventional scavengers and clinical drugs. In a murine model of IRI, it markedly reduced infarct volume by 67%, improved neurological function, and decreased neuronal apoptosis. Additionally, the system provides contrast−enhanced magnetic resonance imaging, enabling real−time monitoring and therapeutic evaluation.

##### Photoacoustic Imaging (PAI)

PAI combines the high contrast of optical imaging with the deep tissue penetration of US.^[^ ^187^ ^]^ When biological tissues are irradiated by pulsed laser light, they absorb optical energy, undergo thermoelastic expansion, and emit US waves, which are detected to form an image. The imaging efficacy depends on the light absorption capacity. LDHs−based nanozyme can efficiently load organic dyes (e.g., ICG, IR780), porphyrin−based compounds, or nanomaterials (e.g., Au NPs) with strong NIR absorption, serving as excellent PAI contrast agents. Meanwhile, LDHs−based nanozyme, when incorporated with certain metal ions (such as Fe^3+^), also exhibits intrinsic absorption at specific wavelengths. A key advantage lies in the ability of LDHs−based nanozyme to protect organic dyes prone to photobleaching and degradation, thereby significantly improving stability. Furthermore, LDHs−based nanozyme facilitate high accumulation of contrast agents at tumor sites, generating intense photoacoustic signals.^[^ ^117^ ^]^ This capability further enables the guidance of subsequent PTT.

##### Fluorescence Imaging (FI)

FI utilizes fluorescent probes that emit light upon excitation at specific wavelengths to enable real−time, highly sensitive imaging.^[^ ^188^ ^]^ When fluorescent molecules are excited by light, they emit fluorescence at a longer wavelength.

To enhance FI performance, fluorescent dye molecules (such as FITC or DOX) can be intercalated into or adsorbed onto LDHs−based nanozyme. Furthermore, the layered structure of LDHs−based nanozyme can isolate dye molecules prone to aggregation−caused quenching (ACQ), preventing their face−to−face stacking and subsequent fluorescence quenching, thereby considerably enhancing fluorescence intensity through a dequenching effect. This strategy improves the biocompatibility and stability of the dyes. It also enables real−time tracking of drug delivery and release processes. For example, the chemotherapeutic agent DOX exhibits intrinsic fluorescence. When loaded into LDHs−based nanozyme, it allows visual monitoring of intracellular drug distribution.

##### Computed Tomography (CT)

CT imaging relies on high atomic number (High−Z) elements to strongly absorb X−rays and generate contrast.^[^ ^189^ ^]^ Elements such as iodine (I), barium (Ba), Au, and gadolinium (Ga) exhibit significantly greater X−ray absorption capabilities compared to soft tissues.^[^ ^189^ ^]^ Loading iodine−based contrast agents (e.g., iohexol) or Au NPs onto LDHs−based nanozyme enables high payloads of High−Z elements and promotes tumor−targeted delivery. This strategy enhances both the contrast efficacy and accuracy of CT imaging. Wang et al.^[^ ^190^ ^]^ developed a Gd−doped MgAl–LDH/Au nanocomposite for computed tomography (CT) and magnetic resonance (MR) dual−modal imaging and drug delivery. By leveraging the layered structure and high specific surface area of LDHs, the material achieved a high doxorubicin (DOX) loading capacity of 264 mg g^−1^. Furthermore, surface−modified Au nanoparticles significantly enhanced the CT imaging performance, yielding a Hounsfield unit (HU) value 2.5 times higher than that of clinical contrast agents. The Gd^3+^ doped within the LDHs framework exhibited a high accessibility, leading to a superior T_1_ relaxivity of 6.6 mM^−1^s^−1^ compared to commercial contrast agents. The nanocomposite enabled intelligent DOX release in the acidic tumor microenvironment and efficient cellular uptake via clathrin−mediated endocytosis, synergistically integrating imaging and therapeutic functions to markedly improve antitumor efficacy. This study highlights the critical role of LDHs−based structures in theranostic platforms.

## 4 Perspective and Conclusions

In this comprehensive review, we have systematically summarized the advances in designing LDHs−based nanozyme for precisely reprogramming redox homeostasis and the pathological microenvironment of anti−inflammatory and pro−inflammatory therapy. We first focuse on structural engineering strategies, including exfoliation, intercalation, loading, alloying, and the formation of mixed metal oxides, which highlighting how these approaches are crucial for tailoring the composition, structure, and ultimately enzyme−mimicking functions of these versatile materials. Subsequently we elucidate the the multi−enzyme catalytic activities of LDHs−based nanozyme, such as CAT−, POD−, OXD−, and SOD−like activity, with a detailed analysis of the catalytic pathways and underlying molecular mechanisms. A key feature is the programmable multi active site configuration of LDHs−based nanozyme, which distinguishes them fundamentally from other catalytic nanomaterials. This distinctive characteristic, accomplished through precise structural control including cation regulation, vacancy engineering, and interlayer modification, enables the deliberate integration of diverse enzymatic activities. Such design flexibility allows creation of custom built nanozymes capable of adapting to and remodeling complex pathological microenvironments, achieving a level of functional integration and responsive control largely beyond the reach of conventional single function or fixed activity nanozyme systems. These activities enable the precise modulation of redox homeostasis under pathological conditions by managing ROS levels and intervening in energy metabolism. Finally, we comprehensively illustrated how structurally engineered LDHs−based nanozyme, as a versatile platform technology, has demonstrated significant therapeutic efficacy not only in cancer and IRI but also hold promise for a wider spectrum of redox−related diseases.

Nevertheless, the multifunctional nature of LDHs−based nanozyme retains significant potential for further development and application. Structurally, the relatively rigid crystalline framework facilitates structure–activity relationship studies but also restricts structural flexibility and the diversity of active sites.^[^ ^85^ ^]^ Although strategies such as intercalation chemistry^[^ ^191^ ^]^ and metal cluster loading^[^ ^42^ ^]^ offer partial mitigation, it remains insufficient to achieve the high atom utilization efficiency and superior catalytic performance^[^ ^192^ ^]^ exhibited by single−atom catalysts. Therefore, the pursuit of highly efficient active sites continues to be a critical and ongoing research direction.

Another significant challenge involves the unstable nature of the structural memory effect in some LDHs systems. This effect, which allows reconstructed layered structures to regain their original configuration, has attracted considerable research interest. However, many LDHs materials lose this capability when exposed to high temperatures, resulting in separated phases or unevenly distributed components. This inconsistency directly affects catalytic performance and makes experimental results difficult to reproduce. Future investigations should focus on developing better observation techniques that can track structural changes in real time at the atomic scale. Simultaneously, researchers should explore new methods to stabilize LDHs structures, potentially through careful addition of other elements, surface modification techniques, or creating confined environments. Solving these fundamental questions will advance our understanding of LDHs transformation processes and enable the development of more reliable LDHs−based nanozyme for medical applications.

Moreover, LDHs−based nanozyme exhibits inferior targeting capabilities compared to the organic counterparts.^[^ ^193^ ^]^ Future research should prioritize the development of organic–inorganic hybrid systems to improve targeted delivery and biocompatibility. Additionally, the inherent alkaline property of LDHs−based nanozyme causes degradation under weakly acidic conditions, leading to the release of metal ions.^[^ ^194^ ^]^ While this ion release may partially modulate the microenvironment, excessive accumulation raises biosafety concerns, and thorough toxicological evaluations are still required. Furthermore, LDHs−based nanozyme often possesses multiple enzyme−mimetic activities, which can result in complex and competing catalytic reactions.^[^ ^161^ ^]^ For example, Fe^2+^, known for Fenton−like activity, frequently engages in parallel catalytic processes that may involve mutual inhibition or trade−offs among different pathways, thereby reducing the controllability and predictability of therapeutic outcomes. Thus, elucidating the underlying catalytic mechanisms and balancing multi enzyme−mimicking activities remain imperative challenges for advancing precision medicine applications.

Owing to the tunable metal ion composition and variable interlayer structure, LDHs−based nanozyme possesses attractive physicochemical properties, diverse enzyme−like activities, and excellent drug loading/delivery capabilities. These characteristics make it highly promising for modulating redox homeostasis to reprogram pathological microenvironments. Despite significant progress, the potential of LDHs−based nanozyme remains underexplored. To facilitate clinical translation, the biodistribution, metabolic pathways, and degradation mechanisms of LDHs−based nanozyme must be thoroughly investigated and evaluated, particularly given multi−elemental.

## Conflict of Interest

The authors declare no conflict of interest.
